# Size exclusion chromatography based proteomic and degradomic profiling of inflammasome-activated, murine bone marrow-derived dendritic cells highlights complex retention and release of cleavage products†

**DOI:** 10.1039/d4mo00163j

**Published:** 2024-10-02

**Authors:** Daniel Vogele, Svenja Wöhrle, Benedikt S. Saller, Klemens Fröhlich, Bálint András Barta, Miguel Cosenza-Contreras, Olaf Groß, Oliver Schilling

**Affiliations:** a Institute for Surgical Pathology, Medical Center – University of Freiburg, Faculty of Medicine, University of Freiburg 79104 Freiburg Germany oliver.schilling@uniklinik-freiburg.de +49 761 270-80610; b Faculty of Biology, University of Freiburg 79104 Freiburg Germany; c Institute of Neuropathology, Faculty of Medicine, Medical Center, University of Freiburg 79106 Freiburg Germany; d Signalling Research Centres BIOSS and CIBSS, University of Freiburg 79106 Freiburg Germany; e Scientific Research Laboratory, Heart and Vascular Center, Semmelweis University Budapest Hungary

## Abstract

Coupling size exclusion chromatography (SEC) with mass spectrometry-based proteomics enables investigating protein complexes, with degradomic profiling providing deeper insights into complex-associated proteolytic processing and retaining of cleavage products. This study aims to map protein complex formation upon inflammasome activation in bone marrow-derived dendritic cells (BMDCs) from gasdermin D-deficient mice, focusing on proteolytic enzymes and truncated proteins in higher molecular weight complexes. Cultured BMDCs were primed with LPS and subsequently treated with nigericin or Val-boroPro (VbP). SEC-fractionated proteins were TMT-labelled and analyzed *via* liquid chromatography-tandem mass spectrometry (LC-MS/MS). We identified 6862 proteins and 70 802 peptides, including 14 714 semi-tryptic peptides indicating elevated endogenous proteolytic processing. The sequence motif of numerous cleavage sites maps to caspase-like activity. Inflammasome activation was corroborated by elevated levels of apoptosis-associated speck-like protein containing a CARD (ASC) in higher molecular weight (MW) fractions and increased IL-1β levels in low MW fractions upon nigericin or VbP treatment. The majority of truncated cleavage products remained within their corresponding, higher MW protein complexes while caspase-specific cleavage products of Rho-associated protein kinase 1, gelsolin, and AP-2 complex subunit alpha-2 dissociated to lower MW fractions. SEC profiles identified 174 proteases, with cell surface proteases forming high MW complexes, including ADAMs and DPP4 but not MMP14. VbP treatment led to the accumulation of ISG15 in low MW fractions while RNA polymerase II coactivator p15 shifted to higher MW fractions. This study demonstrates that SEC-coupled proteomics and degradomic profiling offer unique insights into protein complex dynamics and proteolytic processes upon inflammasome activation.

## Abstract 2

SEC-coupled proteomics and degradomic profiling uncover inflammasome activation-induced protein complex dynamics, proteolytic cleavage events, and provide a global overview of protease SEC profiles in gasdermin D-deficient BMDCs.

## Graphical abstract

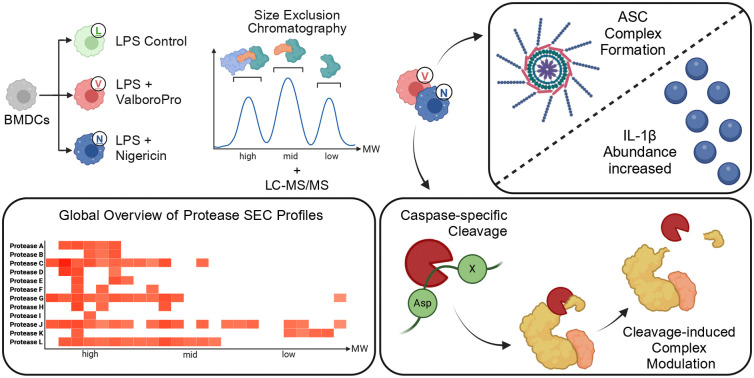

## 1. Introduction

Protein complexes are fundamental to a wide array of cellular processes and have significant roles in both health and disease. These complexes are highly dynamic, adjusting their composition and interactions in response to cellular conditions and environmental stress. Understanding how these complexes function, especially under stress conditions like inflammation, is essential for elucidating their biological functions and implications in disease mechanisms.

Traditionally, the study of protein complexes has utilized methods such as co-immunoprecipitation and gel electrophoresis.^1,2^ However, the advent of advanced mass spectrometry-based techniques has significantly enhanced our capability to analyze these complexes in greater detail. These newer methods reveal the dynamic nature of protein complexes as they change in response to physiological conditions and treatments. A significant advancement in this field is deep interactome profiling by mass spectrometry (DIP-MS), which combines affinity purification with computational analytics to provide detailed insights into protein interactions and complex formations.^3^

Proteolytic processing plays a pivotal role in signaling cascades, particularly in the context of inflammation. Analyzing proteolytic products, their cleavage patterns, the responsible proteases, and their behaviors within a biological setting is crucial for understanding the underlying mechanisms of biological processes. Degradomic profiling, which analyzes proteases and the proteolytic products of their substrates on a system-wide scale, provides a comprehensive perspective by identifying cleavage patterns and substrates critical for the regulatory mechanisms driving inflammatory responses. Chemical enrichment strategies, such as N-TAILS, C-TAILS and HUNTER are typically used to probe deeper into these proteolytic products, enhancing the depth of analysis.^4–6^ Recent advancements in both sensitivity and throughput of liquid chromatography (LC)-tandem mass spectrometry MS/MS instrumentation have also facilitated the use of bioinformatic tools that can analyze proteolytic patterns directly, without the need for prior biochemical enrichment.^7,8^

This study leverages size exclusion chromatography (SEC) combined with proteomic and degradomic profiling to explore the dynamics of protein complexes and their proteolytic processing in the context of an *ex vivo* model for inflammation. We focus on the dynamic state of these complexes in bone marrow-derived dendritic cells (BMDCs) treated with lipopolysaccharide (LPS), and further investigate the effects of subsequent treatments with nigericin or Val-boroPro (VbP, also known as talabostat or PT-100). LPS, a TLR4 agonist, activates pathways such as NF-κB and mitogen-activated protein kinase (MAPK), initiating the transcription of pro-inflammatory genes. Especially in inflammasome competent cells, such as BMDCs, the NF-κB activation results in the activation of transcription factors and the production of pro-inflammatory cytokines such as pro-IL1β and chemokines.^9,10^ In inflammasome activation, this is acknowledged as a priming step. Actual inflammasome activation is triggered by diverse danger signals, also known as pathogen associated molecular patterns (PAMPs) or danger associated molecular patterns (DAMPs). A classical NLRP3 inflammasome activator is the ionophore nigericin, which disrupts cellular ion gradients, particularly affects potassium and proton gradients, leading to activation of the NLRP3 inflammasome and subsequent processing and release of inflammatory cytokines such as IL-1β. Nigericin is often used to induce inflammatory responses in experimental settings.^11,12^ Further, the dipeptidyl peptidase inhibitor VbP has been demonstrated to be a sufficient NLRP1 inflammasome activator.^13^ VbP stimulates cytokine and chemokine production through IL-1β-driven autocrine and paracrine pathways in immune and stromal cells. The assembly of the NLRP3 and NLRP1 inflammasome result in caspase-1 activation. Active caspase-1 is crucial for the conversion of proIL-1β in its bioactive form.^14–16^

In the present study, we investigate the landscape and dynamic rearrangement of proteins and proteolytically truncated cleavage products in a BMDC model for inflammasome activation.

## 2. Materials and methods

### 2.1. Mice

20 to 40-week-old male and female *GSDMD*^−/−^,^17^*Pycard*^−/−^,^18^*Casp1*^−/−^,^17^ and wild-type mice on C57BL/6 background were housed under SOPF or SPF conditions at the Center for Experimental Models and Transgenic Services (CEMT, Freiburg, Germany), the Zentrum für Präklinische Forschung (ZPF, Munich, Germany), in accordance with local guidelines.

### 2.2. Bone-marrow derived dendritic cells preparation and stimulation for MS and inflammasome stimulation

BMDCs were differentiated from tibial and femoral bone marrow as described in detail.^19^ Recombinant murine GM-CSF from Immunotools was used at 20 ng mL^−1^. After seven days of differentiation, cells detached and 20 × 10^6^ cells were seeded in 10 cm dishes or 8–10 × 10^4^ cells in 96-wells, respectively. Inflammasome activation was done as described in detail.^20^ Cells were primed with 50 ng mL^−1^*E. coli* K12 ultra-pure LPS (InvivoGen) for 3 h, followed by treatment with 5 μM nigericin (Sigma-Aldrich) for 1 h (for MS) or 4 h (for inflammasome activation) or 10 μM Val-boroPro (MedChem Express) for 10 h. MG132 (Sigma-Aldrich) was used from 50–200 nM. Experiments were performed in triplicates.

### 2.3. Cell viability assays

Lytic cell death was determined by measuring Lactate-dehydrogenase (LDH) release from cell-free supernatants using a colorimetric assay (Promega, Takara) according to the manufacturer's protocol. Medium served as blank value and was subtracted from the sample values. Results were plotted as percentage of 100% dead cells lysed with lysis buffer 45 min prior to collection of the cell supernatants. Data is depicted as mean ± SD of technical triplicates.

### 2.4. Immunodetection of proteins

Mature IL-1β (p17) was quantified in cell-free supernatants by ELISA (Thermo Fisher). ELISA data is depicted as mean ± SD of technical triplicates as previously described.^19^ For immunoblot analysis, cell-free supernatant and cell lysate samples in SDS- and DTT-containing sample buffer were analyzed. Triplicates were pooled and proteins were separated by SDS-PAGE and transferred to nitrocellulose membranes using standard techniques.^19^ The following primary antibodies were used: anti–caspase-1 (p20) monoclonal antibody mAb (Caspase-1, Adipogen, no. AG-20B-0042-C100, PRID: AB_2755041), IL-1β/IL-1F2 pAb (R&D Systems, no. AF-401-NA, PRID: AB_416684), anti-ASC pAb (AL177, Adipogen, no. AG-25B-0006TS, RRID:AB_2490442), anti-GSDMD mAb (EPR19828, Abcam, EPR19828, Abcam, no. ab209845, PRID:AB_2783550), β-actin mAb (8H10D10, no. 3700, Cell Signaling Technology, no. 3700, RRID:AB_2242334).

### 2.5. Cell harvest and fractionation by size exclusion chromatography

BMDCs stimulated as described in Section 2.3 were harvested and washed four times with ice-cold phosphate-buffered saline (PBS). Cells were resuspended in 2 mL ice-cold PBS, containing PhosSTOP^TM^ (Sigma-Aldrich), PMSF (Sigma-Aldrich) and E64d (Sigma-Aldrich). Cell lysis was performed exclusively using ultrasonication with a Bioruptor for 20 cycles (40 seconds on, 30 seconds off) at a high intensity setting. Following lysis, the mixture was centrifuged for 15 minutes at 15 000×*g*. The resulting supernatant was then loaded onto an ÄKTA HPLC system equipped with a BioSep 5 μm SEC-S4000 500 Å, 600 × 7.8 mm LC column with a flow rate of 750 μL min^−1^. Fraction collection was started 5 minutes after injection, with all fractions being initially collected. For further processing, 21 SEC fractions were selected based on their overlapping elution profiles, indicating uniform protein peaks across the nine samples processed.

### 2.6. Proteomic sample preparation

From each SEC fraction, 100 μg of protein underwent reductive alkylation using 5 mM DTT and 25 mM IAA in a PBS and 200 mM HEPES buffer. Tandem mass tag (TMT) reagents were added to all samples according to the Table S1 (ESI†). Briefly, fraction 01 from the nine samples was combined into one TMT 11-plex set, which included a reference channel (comprising a mix of all samples before SEC) and an empty channel. Similarly, fraction 02 from each sample was processed in the same manner, with TMT channels rotated throughout the fractions. Alongside the TMT incubation, tryptic digestion was carried out in two stages: first, 2.5 μg of trypsin was added and the mixture was incubated for 2 hours at 42 °C; then, after adding another 2.5 μg of trypsin, it was incubated overnight at 37 °C. After overnight digestion, all samples were combined according to their fraction number. Trifluoroacetic acid (TFA) was added to adjust for pH 2 and samples were filtered on 10 kDa MWCO. The flowthrough was diluted in high pH buffer A (10 mM ammonium formate, pH 10) and pH adjusted to pH 10 using 10 mM NaOH. Peptides were injected *via* a 40 μL sample loop multiple times on the Agilent 1100 HPLC system fitted with a XBRIDGE® Peptide BEH C18, Agilent column (3.5 μm, 130 Å, 1 × 150 mm), operating at 40 μL min^−1^. A 60 min gradient from 10 mM ammonium formate (pH 10.0) to 10 mM ammonium formate (pH 10.0), 70% acetonitrile was performed. 50 high pH fractions were collected and concatenated into five fractions submitted to MS.

### 2.7. Mass spectrometry

For MS measurement, vacuum dried peptides were solubilized in 0.1% (v/v) formic acid, sonicated for 5 min and centrifuged at 20 000×*g* for 10 minutes before transferring the supernatant to a measurement tube. 800 ng of each sample, together with 200 fmol of indexed retention time (iRT) peptides, were analyzed as previously described,^21^ using a Q-Exactive plus (Thermo Fisher Scientific) coupled to a nanoflow liquid chromatography (LC) system Easy-nLC 1000 (Thermo Fisher Scientific). The mass spectrometer was operated in data dependent acquisition mode and each MS scan was followed by a maximum of 10 MS/MS scans (Top10 method). The mass range from 300 to 2000 *m*/*z* (mass-to-charge ratio) was analyzed. MS1 resolution was set to 70 000, automatic gain control (AGC) to 3e6 and maximum injection time was set to 50 ms. MS2 resolution was set to 35 000, AGC to 1e5 and maximum injection time to 100 ms using stepped normalized collision energy (NCE) of 32 for fragmentation.

### 2.8. Data analysis

Raw data obtained from the mass spectrometry was analyzed using FragPipe pipeline (v20.0)^22–24^ with a mouse proteome database containing Uniprot sequences downloaded from Uniprot on 6th November 2023 (21 518 entries). Decoys and contaminants for the database search were added using the implemented function in the FragPipe database section. A precursor mass tolerance of 10 ppm and fragment mass tolerance of 20 ppm was used. Semi-specific tryptic cleavage specificity with one missed cleavage was applied. Carbamidomethyl at cysteines as well as a TMT mass delta of 229.16293 at lysines was set as fixed modifications, whereas N-terminal acetylation and N-terminal TMT mass delta were set as variable modifications.

FragPipe output files were further processed and analyzed using R (v4.1.0) within Rstudio. R. Briefly, PSM files from FragPipe were processed using a script inspired by Clark, David J *et al.*,^24^ mimicking TMT-Integrator. Here peptide-spectrum-matches (PSM) were filtered *e.g.* for >50% purity and reference normalization using the reference channel, containing proteins from all fractions and treatment conditions, was applied. Resulting files were further processed using a script based on the TermineR method for extracting endogenous proteolytic processing information from shotgun proteomics data^7^ and modified it for this approach. The correct TMT-channel assignment was validated by comparing iRT-intensities as an internal labeling control. Principal component analysis (PCA) and partial least squares discriminant analysis (PLS-DA) were performed using the mixOmics package (v6.26.0). Identified peptides were annotated using the Fragterminomics package (v0.2.2) and differential abundance analysis of semi-specific peptides was conducted using limma (v3.58.1). Cleavage patterns of differentially abundant peptides were extracted and visualized using R package pheatmap (v1.0.1). Further visualizations were performed using R package ComplexHeatmap (v2.18.0).

## 3. Results and discussion

### 3.1. Inflammasome activation in BMDCs

**Fig. 1:**
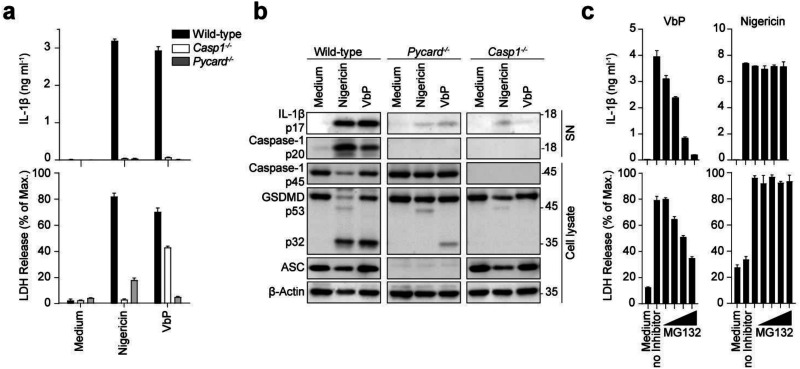
Inflammasome activation by nigericin and Val-boroPro (VbP). (a) and (b) LPS-primed wild-type, ASC (*Pycard*^−/−^) and caspase-1-deficient (*Casp1*^−/−^) BMDCs were stimulated with 5 μM nigericin for 4 hours or 10 μM VbP for 10 hours. IL-1β and LDH (a) were quantified from cell-free supernatants by ELISA and a colorimetric assay, respectively. Data are depicted as mean ± SD of technical triplicates and are representative of at least three independent experiments. Cell lysates and supernatants were analyzed by immunoblotting for the indicated proteins (b). Blots are representative of at least three independent experiments. (c) LPS-primed wild-type BMDCs were incubated with MG132 (50, 100, 150, and 200 nM) 30 min prior to stimulation with 10 μM VbP for 10 hours or 5 μM nigericin for 4 hours. IL-1β and LDH release was measured and analyzed as in (a).

BMDCs from either wild-type, ASC-deficient (*Pycard*^−/−^) or caspase-1-deficient (*Casp1*^−/−^) mice were primed in LPS (50 ng mL^−1^, 3 h) subsequently treated for inflammasome activation by nigericin (5 μM, 4 h) or VbP (10 μM, 10 h). Inflammasome activation was assessed by the release of mature IL-1β by ELISA and the induction of pyroptosis by measuring LDH release. For both nigericin- and VbP-treated BMDCs, release of mature IL-1β and LDH was detected in the wild-type cells, whereas inflammasome activation was reduced in ASC-deficient, and caspase-1-deficient cells (Fig. 1a and b). Comparison of nigericin and VbP treatment highlights partial gasdermin D maturation upon VbP treatment even in the absence of ASC while both compounds require caspase-1 to this end (Fig. 1a and b). This suggests that VbP is activating an inflammasome-nucleating receptor such as NLRP1 or NLRC4 that contain a caspase recruitment domain (CARD) and can thereby directly engage caspase-1 for partial activation in the absence of ASC.^25^ Caspase-1-deficient cells showed after nigericin treatment a slight increase in cytotoxicity (Fig. 1a) and IL-1β release (Fig. 1b). This can be explained due to the time of stimulation (4 h). After 4 h secondary pyroptosis can be observed in BMDCs, involving the engagement of Caspase-8.^17^ In the following experiments, nigericin treatment was reduced to 2 h to prevent secondary pyroptosis. Proteasome inhibition with MG132 resulted in a dose-dependent reduction in VbP-induced inflammasome activation, while no impact was observed for nigericin-induced inflammasome activation (Fig. 1c). This observation aligns with findings by M.C. Okondo *et al.*,^13^ which suggest that VbP activates the NLRP1 inflammasome, which requires proteasomal degradation, releasing its C-terminus from auto-inhibition for activation.^26^ Inflammasome activation by nigericin and VbP treatment was further validated in LPS-primed gasdermin D-deficient BMDCs (Fig. S1, ESI†). A reduction in full-length caspase-1 and an increase in cleaved caspase-1 were observed in both nigericin- and VbP-stimulated cells. Additionally, IL-1β was upregulated in both treatments, primarily in cell lysates, as expected in gasdermin D-deficient cells.

For SEC proteomic profiling of inflammasome activation by either nigericin or VbP, we focused on GSDMD-deficient murine BMDCs. An overview of the protein and peptide coverage is given below (see corresponding section). In addition to the immunoblotting data, we explored our mass spectrometry data to corroborate the inflammasome activation. Samples fractionated by SEC were analyzed using quantitative proteomics, allowing us to monitor the abundance and behavior of proteins across a gradient of molecular weight. Typically, SEC distribution reflects that high molecular weight proteins and those engaged in complexes are eluted in earlier fractions, whereas later fractions predominantly contain lower molecular weight proteins and those not associated with complexes. SEC chromatograms of the three distinct treatment conditions are shown for representative replicate 2 (Fig. S2, ESI†). The general elution profiles did not change significantly between treatment conditions with one distinct peak in early fractions and two partly overlapping peaks towards later fractions.

**Fig. 2:**
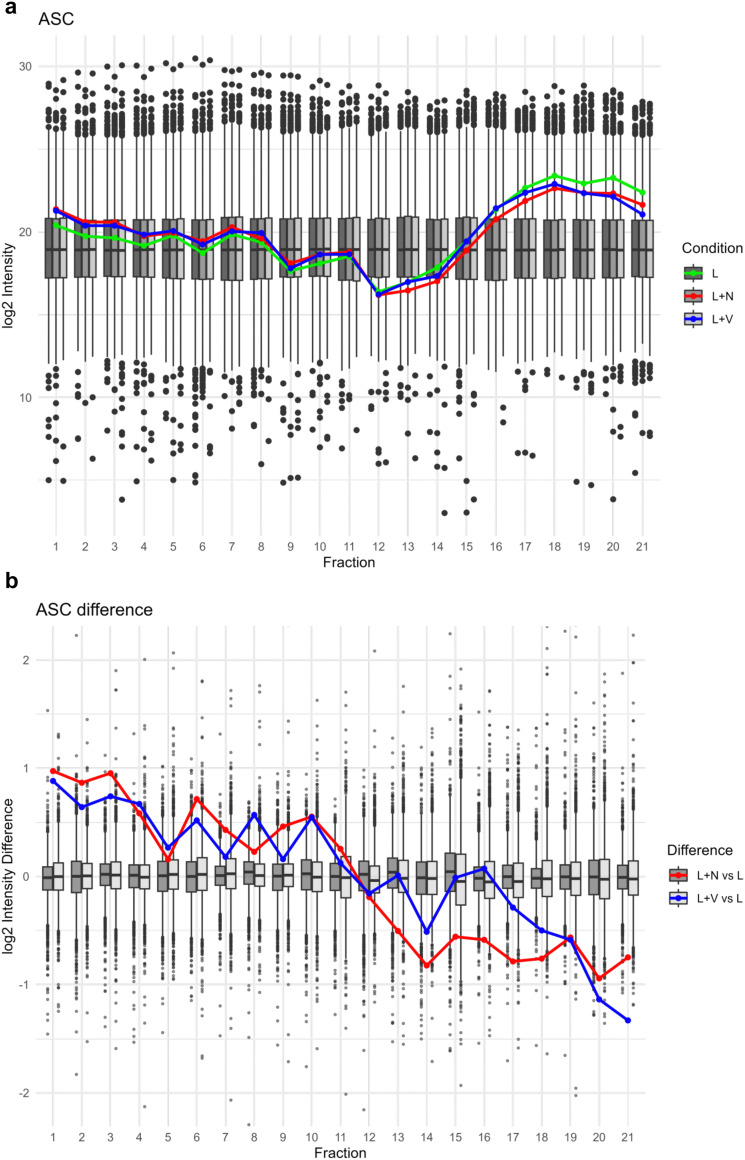
ASC log2 intensity across SEC fractions. (a) Log2 intensity of ASC depicted as line graph for the conditions LPS (L, green), nigericin (L + N, red) and VbP (L + V, blue) in front of boxplot background illustrating the range of abundances for all detected proteins for the conditions LPS (L, dark grey), nigericin (L + N, grey) and VbP (L + V, light grey). (b) Log2 intensity differences of all detected proteins were calculated by subtracting the log2 intensity values of the LPS condition from those of the nigericin and VbP conditions, per protein and per fraction, respectively. Log2 intensity difference of ASC depicted as a line graph for the calculation of nigericin – LPS (L + N *vs*. L, red) and VbP – LPS (L + V *vs*. L, blue) in front of the box plot depiction of the difference range all detected proteins.

ASC displayed elevated abundance in higher molecular weight fractions upon nigericin- or VbP-induced inflammasome activation as compared to LPS-only treatment, suggesting association of ASC as a larger inflammasome protein complex. Conversely, in lower molecular weight fractions, ASC was more prevalent in the LPS-only condition (Fig. 2a and b). This pattern of ASC distribution reinforces the notion of inflammasome activation and polymerization of ASC into filaments^27^ upon NLRP3 or NLRP1 inflammasome when treated with nigericin or VbP.

NLRP1 and NLRP3 inflammasome activation initiates the maturation of pro-caspase-1. Our mass spectrometry analysis revealed no significant variation in the abundance of caspase-1 across different SEC fractions and for the different treatment conditions (Fig. S3a and b, ESI†). Nonetheless, we observed processing of the CARD domain and near the p10 domain of caspase-1 upon nigericin or VbP treatment, suggesting further processing into fully active caspase-1 (Fig. S3c, ESI†).^28^ This would suggest that the active caspase-1 complex, that is thought to be a p20/p10 heterotetramer, has similar migration behavior under our conditions as pro-caspase-1.

**Fig. 3:**
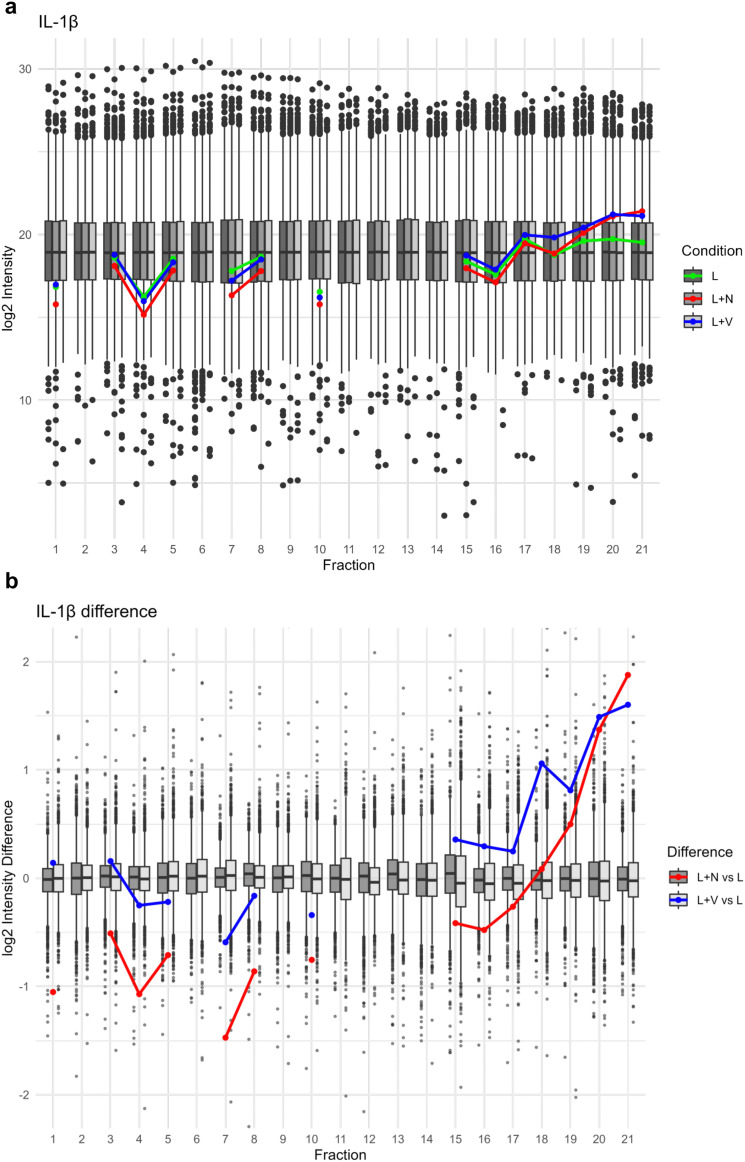
Interleukin-1β log2 intensity across SEC fractions. (a) Log2 intensity of IL-1β depicted as line graph for the conditions LPS (L, green), nigericin (L + N, red) and VbP (L + V, blue) in front of boxplot background illustrating the range of abundances for all detected proteins for the conditions LPS (L, dark grey), nigericin (L + N, grey) and VbP (L + V, light grey). (b) Log2 intensity differences of all detected proteins were calculated by subtracting the log2 intensity values of the LPS condition from those of the nigericin and VbP conditions, per protein and per fraction, respectively. Log2 intensity difference of IL-1β depicted as a line graph for the calculation of nigericin – LPS (L + N *vs*. L, red) and VbP - LPS (L + V *vs*. L, blue) in front of the box plot depiction of the difference range of all detected proteins.

Proteolytic maturation of IL-1β is a hallmark of inflammasome-associated caspase-1 activity. In our SEC proteomics data, we see increased levels of IL-1β in low molecular weight fractions upon inflammasome activation by either nigericin or VbP (Fig. 3); corroborating the prevalence of a shorter, truncated IL-1β form. Notably, the propeptide of IL-1β was not identified, suggesting that it was efficiently processed and likely degraded following cleavage by caspase-1. The increase in mature IL-1β levels, as opposed to the LPS-only stimulated cells, confirms that caspase-1 enzymatic activity is enhanced by treatments with nigericin and VbP, highlighting the functional activation of the inflammasome.

In summary, the results from multiple analytical methods collectively validate the activation of the inflammasome in response to nigericin and VbP treatments. The patterns observed in ASC, caspase-1, and IL-1β across these treatments confirm the functional activity of the inflammasome, highlighting its role in the cellular response mechanisms. Importantly, the use of SEC has effectively illustrated the distributional changes in ASC and (truncated) IL-1β across fractions.

### 3.2. Overview of protein and peptide identifications in proteomic profiling across SEC fractions

General protein and peptide identifications across all 21 SEC fractions showed a high level of detection with counts ranging from 2316 to 3985 proteins and 11 382 to 26 501 peptides (Fig. S4a and b, ESI†). Across all fractions, we identified 6862 proteins.

For each protein, we determined its intact, full-length sequence. The median protein length per SEC fraction was around 500 residues for the 13 SEC fractions, followed by a noticeable decrease (Fig. S4c, ESI†), hinting towards the separation between larger proteins or those involved in complexes, and smaller proteins or those not engaged in complex assembly. Across all fractions, the median protein length across fractions was 472 amino acid residues, further emphasizing the distinct separation in protein size at fraction 14.

In order to additionally identify native and proteolytically generated protein termini, our LC-MS/MS data processing included the identification of semi-specific peptides with TMT-labeled N-termini. Semi-specific peptides are of particular interest in the context of inflammasome complex induced downstream signaling and are in this case defined by their non-trypsin-specific termini, indicative of potential proteolytic activity.

We identified and quantified 6862 proteins and 70 802 peptides with a degradomic coverage of 14 714 semi-specific peptides. As outlined above, semi-tryptic data processing enabled the identification of endogenous, proteolytic cleavage sites in each SEC fraction. To this end, we used the TermineR method to extract proteolytic processing information from shotgun proteomics data.^7^

We aimed at identifying semi-specific peptides that were significantly more abundant for each SEC fraction. We employed linear models of microarray analysis (LIMMA) statistics with an adjusted *p*-value cutoff of 0.05. We focused on comparing either nigericin or VbP treatments *versus* the LPS-only control.

**Fig. 4:**
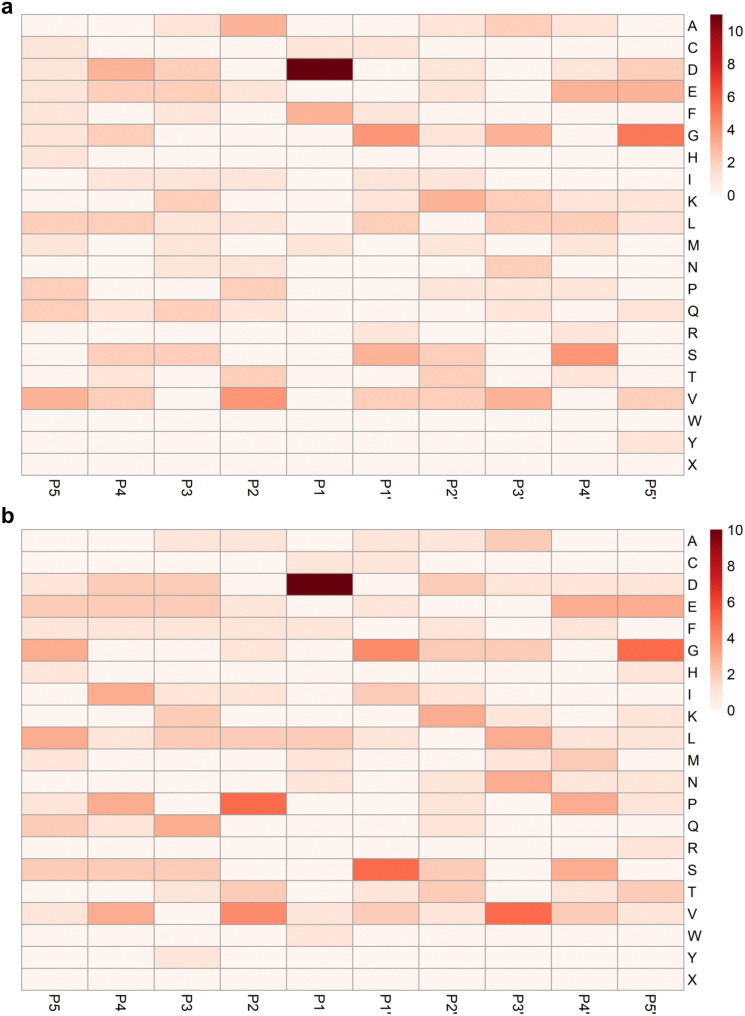
Heatmap depiction of differentially enriched cleavage patterns in representative fraction 7 for nigericin and VbP treatments compared to LPS alone, using an adjusted *p*-value cutoff of 0.05. The color gradient from white to red represents the number of occurrences of each amino acid at the respective position, with darker red indicating a higher number of occurrences. Positional occurrence of amino acids enriched in nigericin (a) and VbP (b) compared to LPS treatment alone was calculated by LIMMA differential abundance analysis utilizing adjusted *p*-value based on semi-specific peptides. Positions are displayed as N-terminal (P5 to P1) and C-terminal (P1′ to P5′) relative to the cleavage site (between P1 and P1′). Amino acids are indicated by their one-letter code.

For this set of induced proteolytic processing sites, we determined the amino acid sequences encompassing the actual cleavage site and depicted them in a heatmap-type approach using the Schechter–Berger nomenclature^29^ (Fig. 4 for the representative SEC fraction 7; Fig. S5 and S6 for all SEC fractions, ESI†). For both nigericin- and VbP-induced inflammasome activation, we noticed the strong presence of a P1D motif in most SEC fractions, often accompanied by a minor enrichment for P4D, P3E or P2V. This cleavage motif is reminiscent of executioner-caspase like activity, *i.e.*, activity of caspases-3, -6, -7 but may also include cleavage events catalyzed by the initiator caspase-1. This set of induced cleavage events includes prototypical substrates of executioner caspases, such as gelsolin, actin, vimentin and myosin-9 (Table S2, ESI†). The prominence of caspase-3, -6 and -7 activity in response to inflammasome activation is consistent with caspase-8 and subsequent caspase-3 activation at ASC specks in the absence of pyroptosis^17^ as well as with the ability of caspase-1 to trigger some caspase-3 activity that is more likely to occur when cell lysis is prevented by GSDMD-deficiency. Such mechanisms are particularly relevant following nigericin and VbP treatments, which are known to induce inflammatory cascades and cellular stress responses. Furthermore, given the stringent cutoff applied, it is plausible that caspase-1 cleavage patterns, although integral to inflammasome activation, are overshadowed by more dominant signals related to other aspects of inflammation and cellular restructuring. Nonetheless, following nigericin and VbP treatments, we observed a significant increase in the abundance of cleavage products from caspase-1 related substrates in our data, such as AP-2 complex subunit alpha-2 (Ap2a2)^30^ (Table S2, ESI†).

Coupling a (bioinformatic) terminomic approach with SEC proteomics enables investigating whether cleavage products show distinguishable SEC profiles as compared to the corresponding proteins (as indicated by fully tryptic peptides). Among the P1D substrates, gelsolin and Ap2a2 showed unique characteristics in their cleaved peptides and general protein abundance across SEC fractions. P1D cleavage patterns were observed across nearly all MW fractions, indicating that truncated cleavage products predominantly remain within their corresponding protein complexes.

**Fig. 5:**
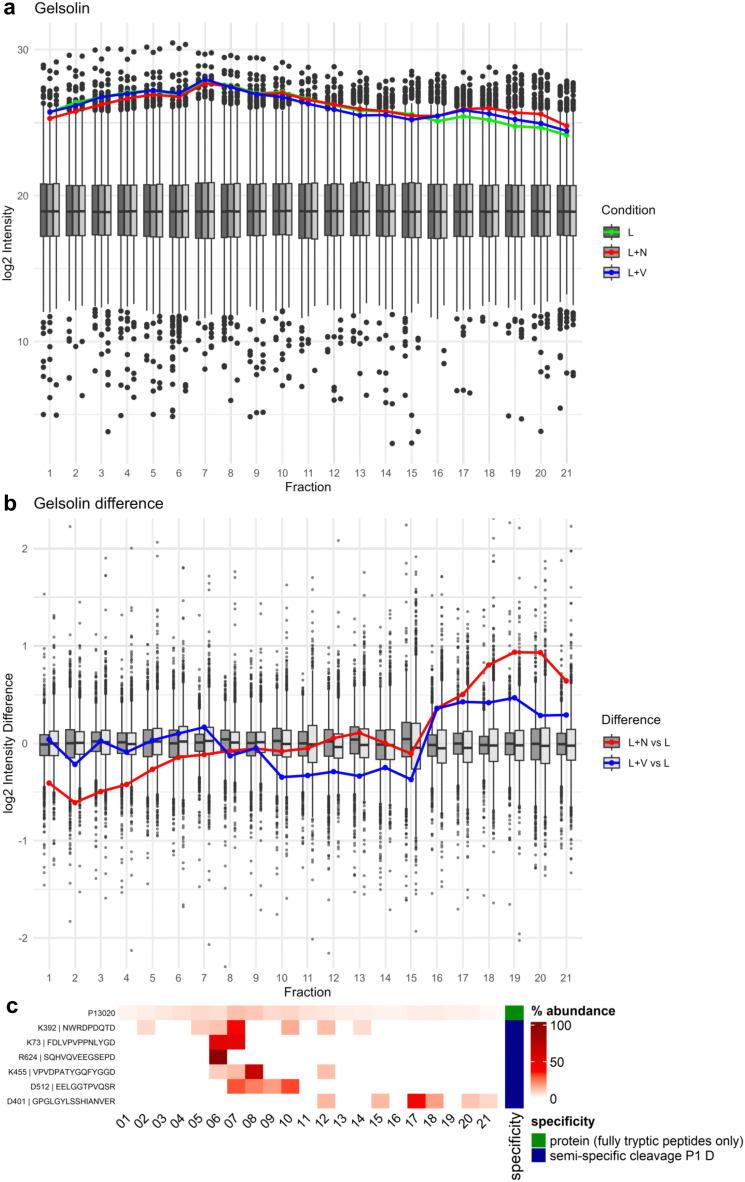
Gelsolin log2 intensity and percentage distribution of peptides across SEC fractions. (a) Log2 intensity of gelsolin depicted as line graph for the conditions LPS (L, green), nigericin (L + N, red) and VbP (L + V, blue) in front of boxplot background illustrating the range of abundances for all detected proteins for the conditions LPS (L, dark grey), nigericin (L + N, grey) and VbP (L + V, light grey). (b) Log2 intensity differences of all detected proteins were calculated by subtracting the log2 intensity values of the LPS condition from those of the nigericin and VbP conditions, per protein and per fraction, respectively. Log2 intensity difference of gelsolin depicted as a line graph for the calculation of nigericin – LPS (L + N *vs*. L, red) and VbP – LPS (L + V *vs*. L, blue) in front of the box plot depiction of the difference range of all detected proteins. (c) Heatmap depiction of percentage distribution of gelsolin protein (P13020) and semi-specific peptides, exemplary for nigericin condition. Raw intensity values were summed per row and percentage contribution per fraction is illustrated by red color gradient. Semi-specific peptides with Asp cleavage are marked as blue and remaining peptides were summarized as tryptic protein in green.

Gelsolin exhibited a pattern of abundance distribution across SEC fractions that suggests its complex disassembly especially upon nigericin treatment, with gelsolin being less abundant in the high molecular weight fractions under nigericin treatment and shifting towards lower molecular weight fractions (Fig. 5a and b). This effect was less pronounced for VbP-induced inflammasome activation.

Gelsolin acts as an actin-binding protein and is crucial for actin filament dynamics by severing and capping actin filaments.^31^ For this reason, we also investigated the SEC profile of actin (Uniprot ID P60710; Fig. S7, ESI†). Actin abundance distributions in SEC aligned with those observed for gelsolin, including the more prominent effect of nigericin treatment with regard to the shift to lower molecular weight SEC fractions. This shift possibly suggests partial actin depolymerization and complex disassembly induced by nigericin, effects that are less pronounced with VbP. The highly similar abundance behaviors of actin and gelsolin raise the hypothesis of their cooperative disassembly. Moreover, the presence of caspase cleavage sites within actin, combined with its known role as a direct caspase substrate, suggests that caspase-mediated depolymerization may also contribute to these changes. Moreover, the caspase-cleaved gelsolin peptide DQTD^401^↓GPGLGYLSSHIANVER eluted in lower MW (predominantly fraction 17) than the bulk of intact gelsolin (Fig. 5c). This suggests that upon caspase activation, gelsolin is cleaved, leading to the detachment of this cleavage fragment from the remaining protein and any potential complex-forming partners. A second caspase-cleaved gelsolin peptide shows an SEC profile similar to gelsolin protein (Fig. 5c). Protein abundances were calculated only on fully tryptic peptides, therefore the higher abundances observed in low MW fraction are based on the shift of intact gelsolin peptides rather than the presence of semi-specific peptides.

**Fig. 6:**
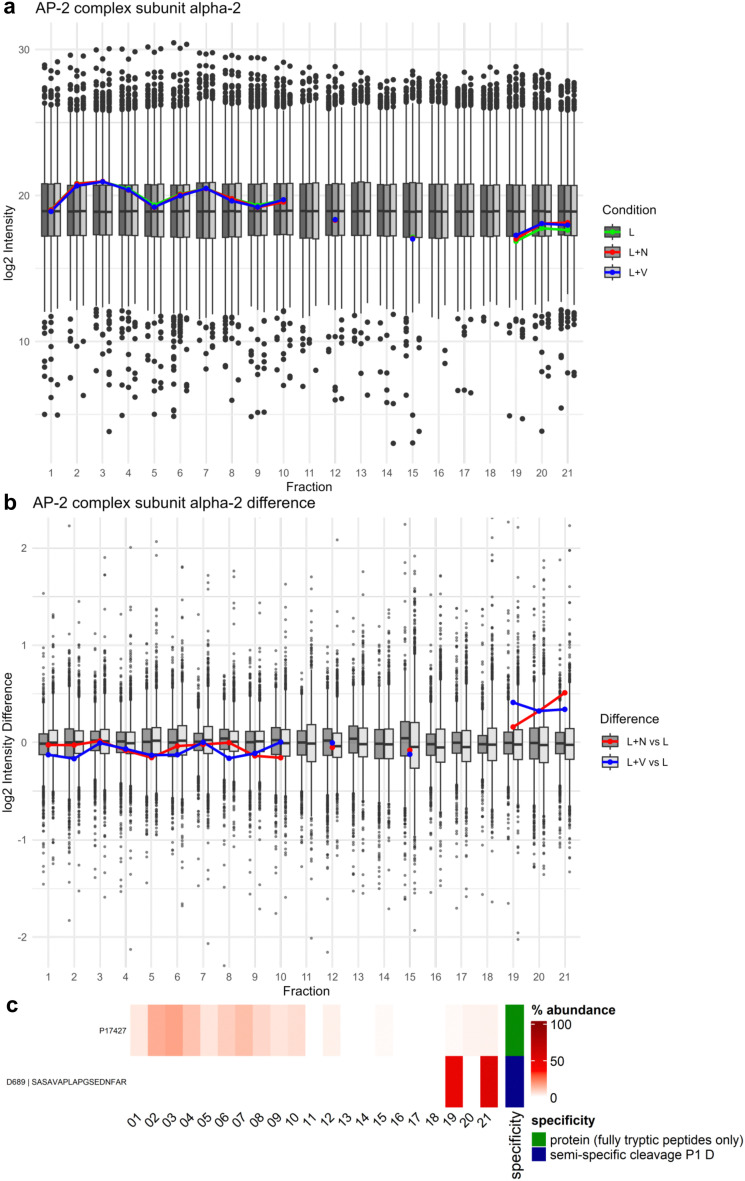
AP-2 complex subunit alpha-2 log2 intensity and percentage distribution of peptides across SEC fractions. (a) Log2 intensity of Ap2a2 depicted as line graph for the conditions LPS (L, green), nigericin (L + N, red) and VbP (L + V, blue) in front of boxplot background illustrating the range of abundances for all detected proteins for the conditions LPS (L, dark grey), nigericin (L + N, grey) and VbP (L + V, light grey). (b) Log2 intensity differences of all detected proteins were calculated by subtracting the log2 intensity values of the LPS condition from those of the nigericin and VbP conditions, per protein and per fraction, respectively. Log2 intensity difference of Ap2a2 depicted as a line graph for the calculation of nigericin – LPS (L + N *vs*. L, red) and VbP – LPS (L + V *vs*. L, blue) in front of the box plot depiction of the difference range of all detected proteins. (c) Heatmap depiction of percentage distribution of Ap2a2 protein (P17427) and semi-specific peptides, exemplary for nigericin condition. Raw intensity values were summed per row and percentage contribution per fraction is illustrated by red color gradient. Semi-specific peptides with Asp in P1 are marked as blue and protein, which only consist of fully tryptic peptides in green.

The alpha subunit of AP-2, such as Ap2a2, plays a role in binding to membrane proteins and selecting them for endocytosis. This complex is also sensitive to changes in cellular signaling and membrane dynamics, which are likely influenced under inflammatory conditions.^32^ For the protein Ap2a2, part of the AP-2 complex involved in clathrin-mediated endocytosis, our mass spectrometry data highlighted consistent presence in higher MW fractions of the SEC profile with no impact by inflammasome activation (Fig. 6a and b). Yet, upon nigericin- and VbP-induced inflammation a higher protein abundance could be observed in low MW fractions and additionally there appeared a dissociated P1D cleavage fragment in lower MW SEC fractions (Fig. 6c).

**Fig. 7:**
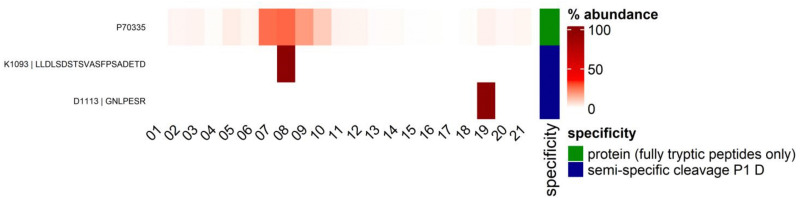
Rho-associated protein kinase 1 (Rock1) percentage distribution of peptides across SEC fractions. Heatmap depiction of percentage distribution of Rock1 protein (P70335) and semi-specific peptides cleaved with aspartate in P1 position, exemplary for nigericin condition. Raw intensity values were summed per row and percentage contribution per fraction is illustrated by red color gradient. Semi-specific peptides with aspartate (D) in P1 are marked as blue and protein, which only consist of fully tryptic peptides in green.

Furthermore, we noticed a P1D cleavage event in Rho-associated protein kinase 1 (Rock1), an effector of the small GTPase Rho, at the position 1113 (Fig. 7). This position is reported to be cleaved by caspase-3 at the conserved DETD^1113^↓G sequence, separating the kinase domain from the autoinhibitory C-terminal coiled-coil domain, resulting in deregulated and constitutively active kinase fragments with a supposed function in the onset of apoptosis.^33,34^ Both N- and C-terminal peptides of this cleavage event were detected in our data (Fig. 7).

The N-terminal peptide (K^1093^↓LLDLSDSTSVASFPSADETD^1113^↓G) was detected in the SEC fraction 8, where the majority of the Rock1 protein elutes. Conversely, the C-terminal peptide (D^1113^↓GNLPESR) eluted later, in fraction 19, indicating the reported separation from the rest of the protein following caspase cleavage. The abundance of this C-terminal peptide in fraction 19 was significantly increased during both nigericin- and VbP-induced inflammasome activation, as determined by LIMMA analysis (Table S3, ESI†). This supports the critical role of Rock1 cleavage under both conditions, which likely contributes to apoptotic downstream events.

Additional proteins were identified which contain caspase-specific cleaved peptides that predominantly elute later in SEC than the majority of the respective whole proteins (Fig. S8a and b, ESI†). Prominent examples include the U2 snRNP-associated SURP motif-containing protein (U2surp) and proliferation marker protein Ki-67 (Mki67). While these proteins did not exhibit general changes in overall abundance distribution and lack a direct, clear connection to inflammation biology, Mki67 demonstrated an increased percentage abundance of the semi-specific peptide in fractions 20 and 21 under nigericin and VbP treatments, suggesting an indirect influence by inflammatory conditions.

### 3.3. Further insight into differential protein complex dynamics *via* SEC proteomics

**Fig. 8:**
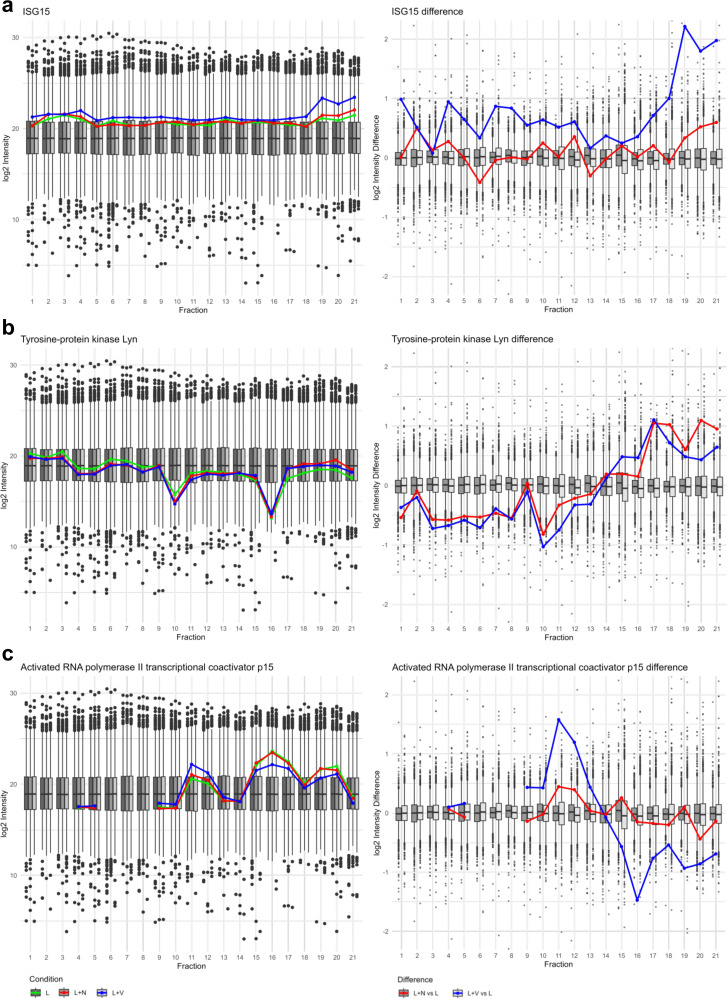
Protein complex dynamics according to log2 intensity distribution and differences across SEC fractions. (left) Log2 intensity distribution of ISG15 (a), tyrosine-protein kinase Lyn (b) and Activated RNA polymerase II trasncribtional coactivator p15 (Sub1) (c) depicted as line graph for the conditions LPS (L, green), nigericin (L + N, red) and VbP (L + V, blue) in front of boxplot background illustrating the range of abundances for all detected proteins for the conditions LPS (L, dark grey), nigericin (L + N, grey) and VbP (L + V, light grey). (right) Log2 intensity differences of all detected proteins were calculated by subtracting the log2 intensity values of the LPS condition from those of the nigericin and VbP conditions, per protein and per fraction, respectively. Log2 intensity difference of ISG15 (a), tyrosine-protein kinase Lyn (b) and Sub1 (c) depicted as a line graph for the calculation of nigericin – LPS (L + N *vs*. L, red) and VbP – LPS (L + V *vs*. L, blue) in front of the box plot depiction of the difference range all detected proteins.

Besides gelsolin, Ap2a2, and Rock1, we identified several other proteins exhibiting signs of complex formation or disassembly in response to nigericin- or VbP-induced inflammasome activation (Fig. 8). Among these, Interferon-stimulated gene 15 (ISG15), was particularly notable. ISG15 is an ubiquitin-like modifier, which conjugates to target proteins through ISGylation. In VbP-induced inflammasome activation (but not for nigericin-induction), ISG15 was found at higher abundance in most SEC fractions, including higher MW fractions, which may indicate elevated ISGylation in addition to a potential pool of elevated, free ISG15 at lower MW fractions. This finding suggests a specific role of ISG15 and ISGylation in VbP-induced inflammasome activation.

The tyrosine-protein kinase Lyn is a negative regulator of toll-like receptor (TLR) signaling and contributes to cytoskeletal rearrangements and actin dynamics.^35,36^ For both nigericin- and VbP-induced inflammasome activation, Lyn was prominently found in late, lower MW fractions suggesting prevalence of free protein and its dissociation from regulatory protein complexes.

Furthermore, the RNA polymerase II transcriptional coactivator p15 (Sub1) displayed an SEC profile indicative of a specific impact by VbP-induced inflammasome activation, with accumulation in mid-range SEC fractions and depletion of free protein from lower MW fractions; pointing towards induced integration into protein complexes.

### 3.4. Protease distribution patterns in SEC proteomics

Based on previous work by Knopf *et al.*,^37^ we aimed to exploit the SEC proteomics data for a global overview of SEC profiles of proteolytic enzymes. In total, our dataset comprised quantitative SEC proteome profiles for 174 proteases (based on protease annotation of MEROPS^38^), including for example, the proteasome, lysosomal proteases, cell surface metalloproteases (*i.e.* A Disintegrin and Metalloproteinases, ADAMs; A Disintegrin and Metalloproteinase with Thrombospondin motifs, ADAMTSs), matrix metalloproteases (MMP), caspases, and dipeptidyl peptidases (DPPs). For SEC profiling, the intensity across all SEC fractions was summed for each detected protease, and the percentage contribution of each fraction to the total sum was calculated (Fig. S9, ESI†).

**Fig. 9:**
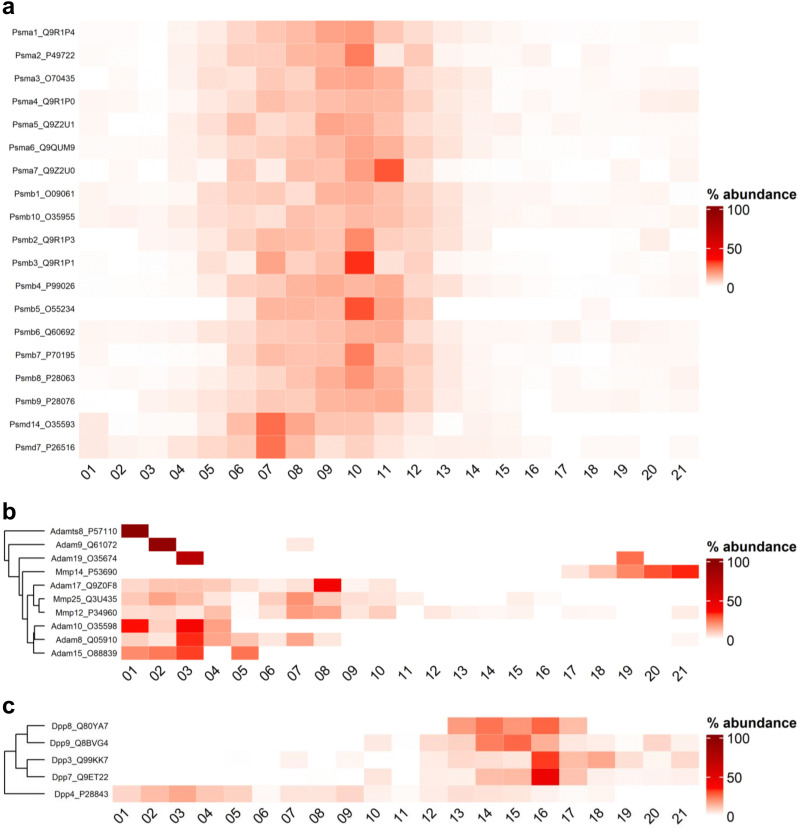
Distribution pattern of selected proteases across SEC fractions. Heatmap depiction of percentage distribution of proteasome (a), ADAMs and MMPs (b) and DPPs (c) across fractions. Raw intensity values were summed per row and percentage contribution per fraction is illustrated by red color gradient from 0–100%. Rows named using the combination of the gene name and Uniprot ID of respective protease. Clustering on rows was performed using “ComplexHeatmap” R-package. Fraction number depicted as *y*-axis.

The proteasome displayed peak intensities at mid-range SEC fractions (Fig. 9a). We detected both the constitutive subunits PSMB 5,6,7 and the immunoproteasome subunits PSMB 8,9,10 with similar SEC profiles.

Most cell surface metalloproteases (ADAMs, ADAMTSs) accumulated in higher MW fractions, indicating their association in multi-protein complexes (Fig. 9b). DPP4 (but not the cytosolic or lysosomal DPPs) displayed a similar SEC profile with strong abundance in higher MW fractions. In earlier work, we have shown that DPP4 is likely present in lipid, together with typical lipid raft components such as caveolin-1.^37^ Consistently, in our SEC analysis, DPP4 shows a distribution pattern similar to lipid raft components including prohibitin, prohibitin-2, transmembrane protein 109, erlin-2, thy-1 membrane glycoprotein, and caveolin-1 (Fig S10, ESI†). Of the cell surface metalloprotease, MMP14 stood out by exclusively eluting in lower MW fractions; also in sharp contrast to the membrane-type MMP25 and MMP12 (Fig. 9b). The mapping of MMP14 was based on three distinct peptides with consistent behavior (Table S4, ESI†). None of the ADAMs or ADAMTSs but all three MMPs were affected in nigericin-induced inflammasome activation but not upon VbP-induction. MMP12 appeared to be down-regulated while MMP14 and -25 are upregulated (Fig. S11, ESI†). The shifted MMP profile is a new aspect of nigericin-induced inflammasome activation.

## 4. Conclusions

This study successfully demonstrates the application of combining size exclusion chromatography (SEC) with proteomic and degradomic profiling to explore protein complex dynamics upon inflammasome activation. Our comprehensive approach enabled the identification and quantification of 6862 proteins and 70 802 peptides, including 14 714 semi-specific peptides, thus providing a detailed overview of the proteome and degradome landscape in BMDCs upon inflammasome activation.

Key features of inflammasome activation were observed, including ASC polymerization and an increased abundance of IL-1β in low molecular weight fractions during both nigericin and VbP treatments. A strong caspase activity fingerprint was observed, highlighted by a significant presence of caspase-like cleavage patterns. This was particularly evident in the cleavage products of Rho-associated protein kinase 1 (Rock1), gelsolin, and AP-2 complex subunit alpha-2 (Ap2a2), which demonstrated distinct profiles of truncation and complex disassembly. The cleavage of Rock1, for instance, resulted in the separation of its regulatory domain, showcasing the dynamic nature of proteolytic processing in response to inflammasome activation. Gelsolin and actin shifted towards lower molecular weight fractions particularly upon nigericin treatment, suggesting their partial depolymerization and complex disassembly. Similarly, AP-2 complex subunit alpha-2 displayed dissociation patterns in lower molecular weight fractions upon inflammasome activation, further illustrating the impact of inflammatory stimuli on protein complex dynamics.

The study offered an overview of protease localization in SEC profiles, identifying 174 proteases with distinct distribution patterns. Notably, cell surface metalloproteases such as ADAMs and DPP4 were predominantly found in high molecular weight fractions, indicating their presence in multi-protein complexes such as lipid rafts. In contrast, MMP-14 uniquely eluted in lower molecular weight fractions with increased abundance in nigericin treatment.

We uncovered features specific to either nigericin- or VbP-induced inflammasome activation. For instance, interferon-stimulated gene 15 (ISG15) showed higher abundance in most SEC fractions during VbP treatment, suggesting a role for ISGylation in this specific inflammatory response. Tyrosine-protein kinase Lyn displayed elevated abundance in low MW fraction in both inflammation treatments while RNA polymerase II transcriptional coactivator p15 is only affected upon VbP treatment, further emphasizing the distinct molecular mechanisms triggered by these two inflammasome activators.

In summary, the integration of SEC with proteomic and degradomic profiling provided unique insights into the complex dynamics of proteins, proteases, and their cleavage products during inflammasome activation.

## Author contributions

Daniel Vogele: investigation, formal analysis, writing – original draft, writing – review & editing, visualization. Svenja Wöhrle: investigation, writing – review & editing. Benedikt S. Saller: investigation, writing – review & editing. Klemens Fröhlich: investigation. Bálint András Barta: investigation. Miguel Cosenza-Contreras: formal analysis, software. Olaf Groß: writing – review & editing. Oliver Schilling: conceptualization, writing – review & editing, supervision, funding acquisition.

## Declaration of generative AI and AI-assisted technologies in the writing process

During the preparation of this work, the authors used ChatGPT for grammar checking and phrase refinement. After using this tool/service, the authors reviewed and edited the content as needed and take full responsibility for the content of the publication.

## Data availability

The raw data used for peptide and protein identification and quantification, as well as the analysis output files and R scripts utilized in this study, are available *via* the Massive database (MassIVE MSV000095490) and the following FTP download link: https://ftp://massive.ucsd.edu/v08/MSV000095490/. Additionally, the code used to annotate and visualize the terminomics data is a modified version of the code available at https://github.com/MiguelCos/Fragterminomics.

## Conflicts of interest

The authors declare no conflict of interest.

## Supplementary Material

MO-020-D4MO00163J-s001

MO-020-D4MO00163J-s002
