# Supplementary material for: Size exclusion chromatography based proteomic and degradomic profiling of inflammasome-activated, murine bone marrow-derived dendritic cells highlights complex retention and release of cleavage products
Source: Mol Omics. 2024 Oct 2;20(9):595–610. doi: 10.1039/d4mo00163j (PMC11460583; doi:10.1039/d4mo00163j)
Supplement: MO-020-D4MO00163J-s001 [file MO-020-D4MO00163J-s001.pdf]

This journal is © The Royal Society of Chemistry 2024

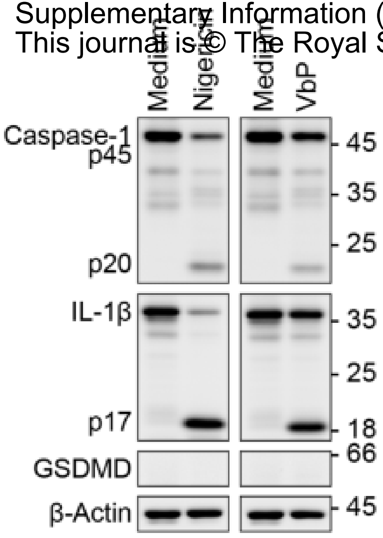

Supplemental figure 1: Inflammasome activation by nigericin and VbP
LPS-primed gasdermin D-deficient (*GSDMD*$^{-/-}$) BDMCs were stimulated with 5 µM nigericin for 1 hours or 10 µM VbP for 10 hours. Cell lysates were analyzed by immunoblotting for the indicated proteins for nigericin and VbP stimulated cells as well as only LPS-primed cells as control (Medium).

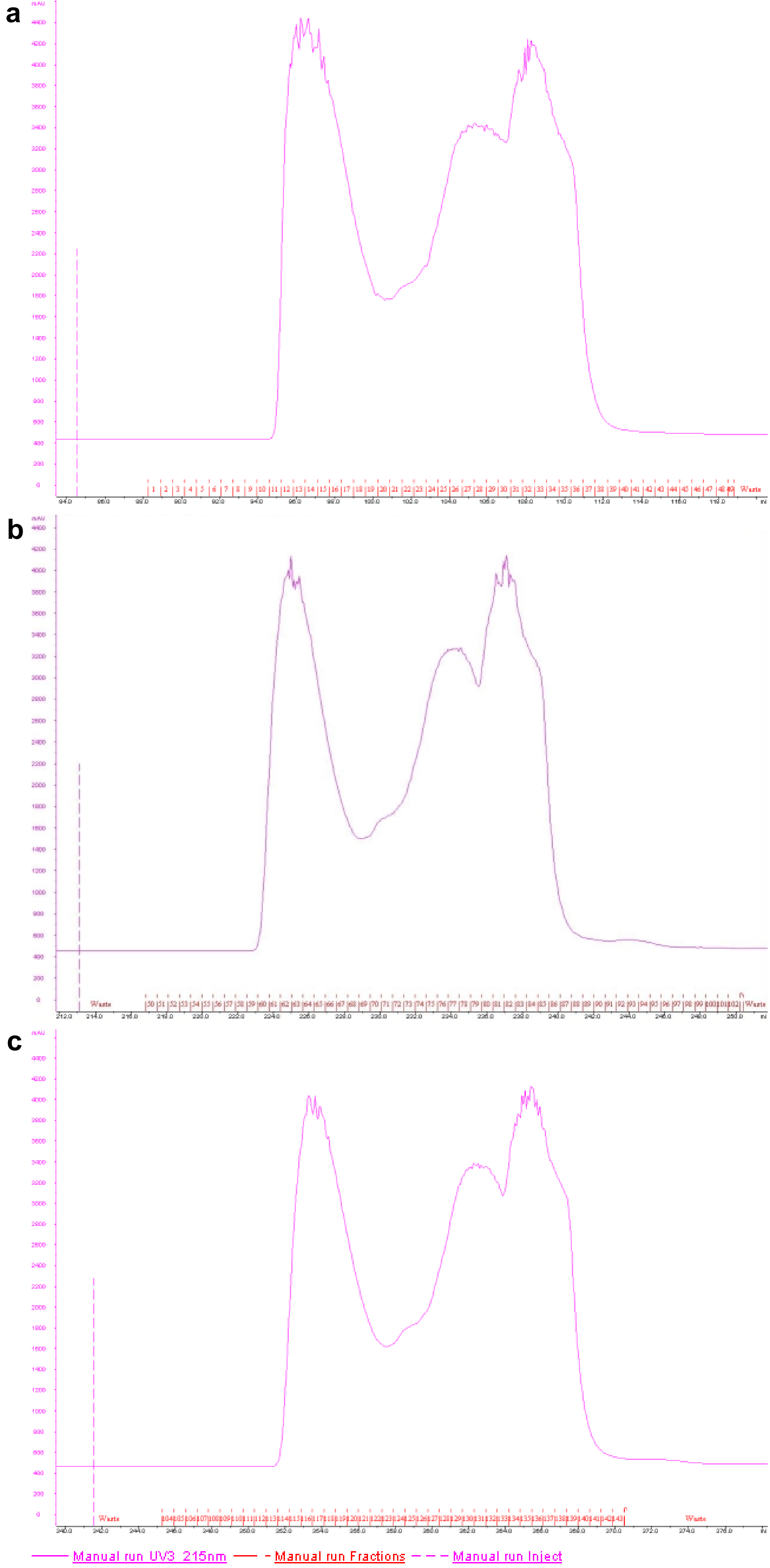

Supplemental figure 2: Size exclusion chromatograms of representative replicate 2 of each treatment condition. Supernatant of lysed cells were loaded onto an ÄKTA HPLC system equipped with a BioSep 5 µm SEC-S4000 500 Å, 600 × 7.8 mm LC column, operating at a flow rate of 750 µl/min. The vertical pink dotted line indicates the sample injection point. All fractions indicated in red were initially collected. Shown are SEC chromatograms of representative replicate 2 for LPS only treatment (**a**), LPS + nigericin treatment (**b**) and LPS + VbP (**c**).

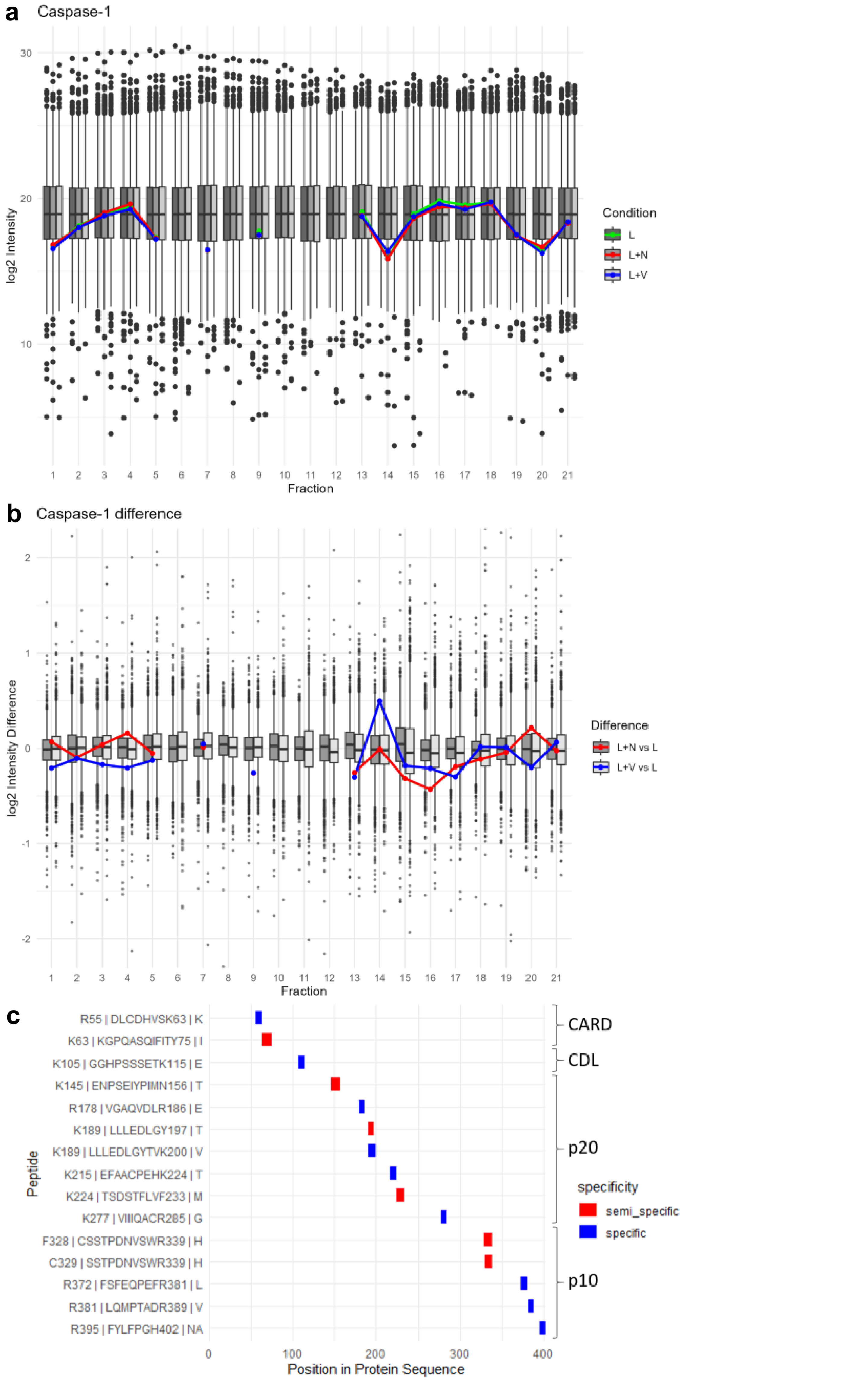

Supplemental figure 3: Caspase-1 log2 intensity across SEC fractions and peptide coverage.
(**a**) Log2 intensity of caspase-1 depicted as line graph for the conditions LPS (L, green), nigericin (L+N, red) and VbP (L+V, blue) in front of boxplot background illustrating the range of abundances for all detected proteins for the conditions LPS (L, dark grey), nigericin (L+N, grey) and VbP (L+V, light grey). (**b**) Log2 intensity differences of all detected proteins were calculated by subtracting the log2 intensity values of the LPS condition from those of the nigericin and VbP conditions, per protein and per fraction, respectively. Log2 intensity difference of caspase-1 depicted as a line graph for the calculation of nigericin - LPS (L+N vs L, red) and VbP - LPS (L+V vs L, blue) in front of the box plot depiction of the difference range all detected proteins. (**c**) Protein coverage with cleavage annotated peptide sequences and annotation of caspase-1 subunits CDL (CARD Domain Linker), p20 (large subunit) and p10 (small subunit).

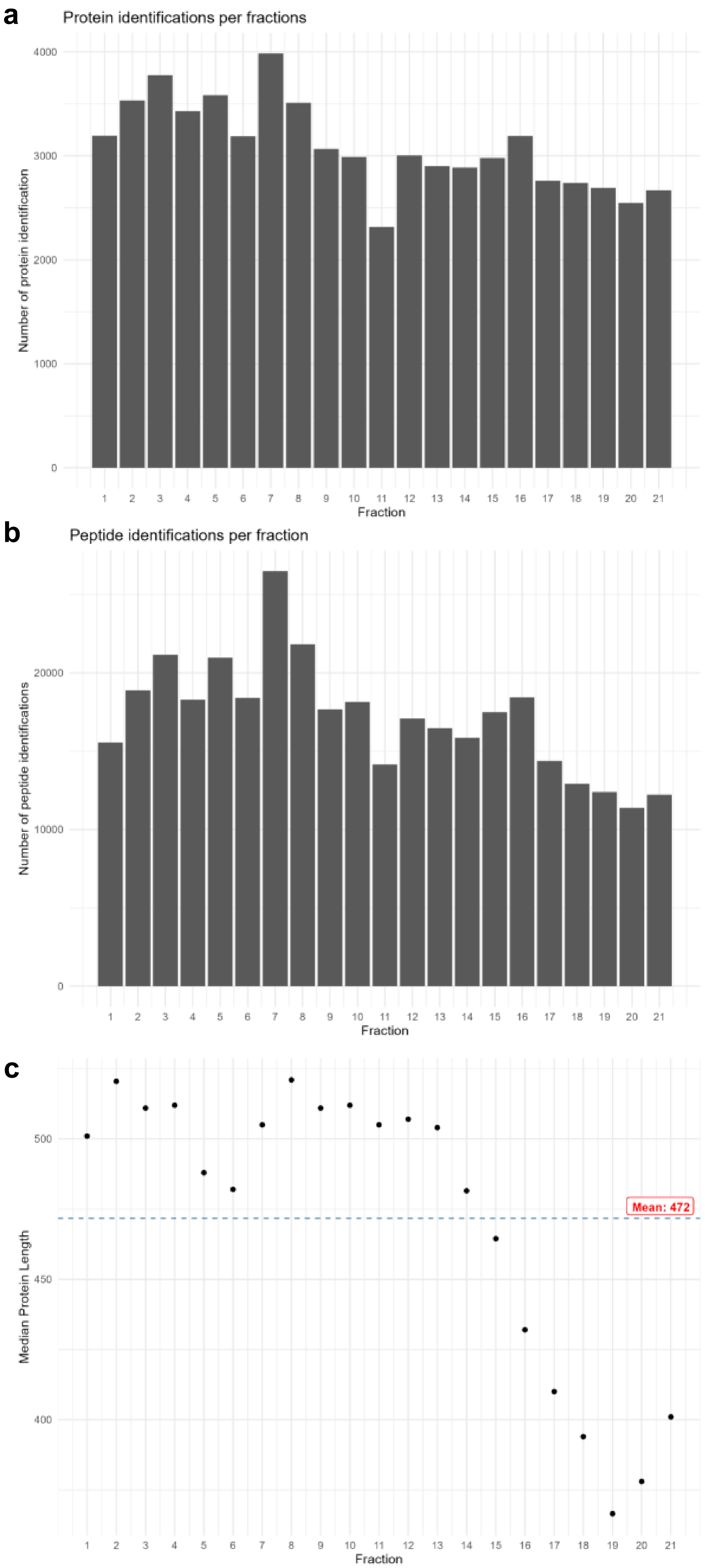

Supplemental figure 4: Overview protein and peptide identifications and distribution of median protein length across SEC fractions.
Total number of proteins (**a)** and peptides (**b**) identified in each of the 21 SEC fractions. (**c**) Changes in median protein length across fractions, illustrating a distinct breakpoint at fraction 14. Mean of median protein length illustrated as dashed blue line.

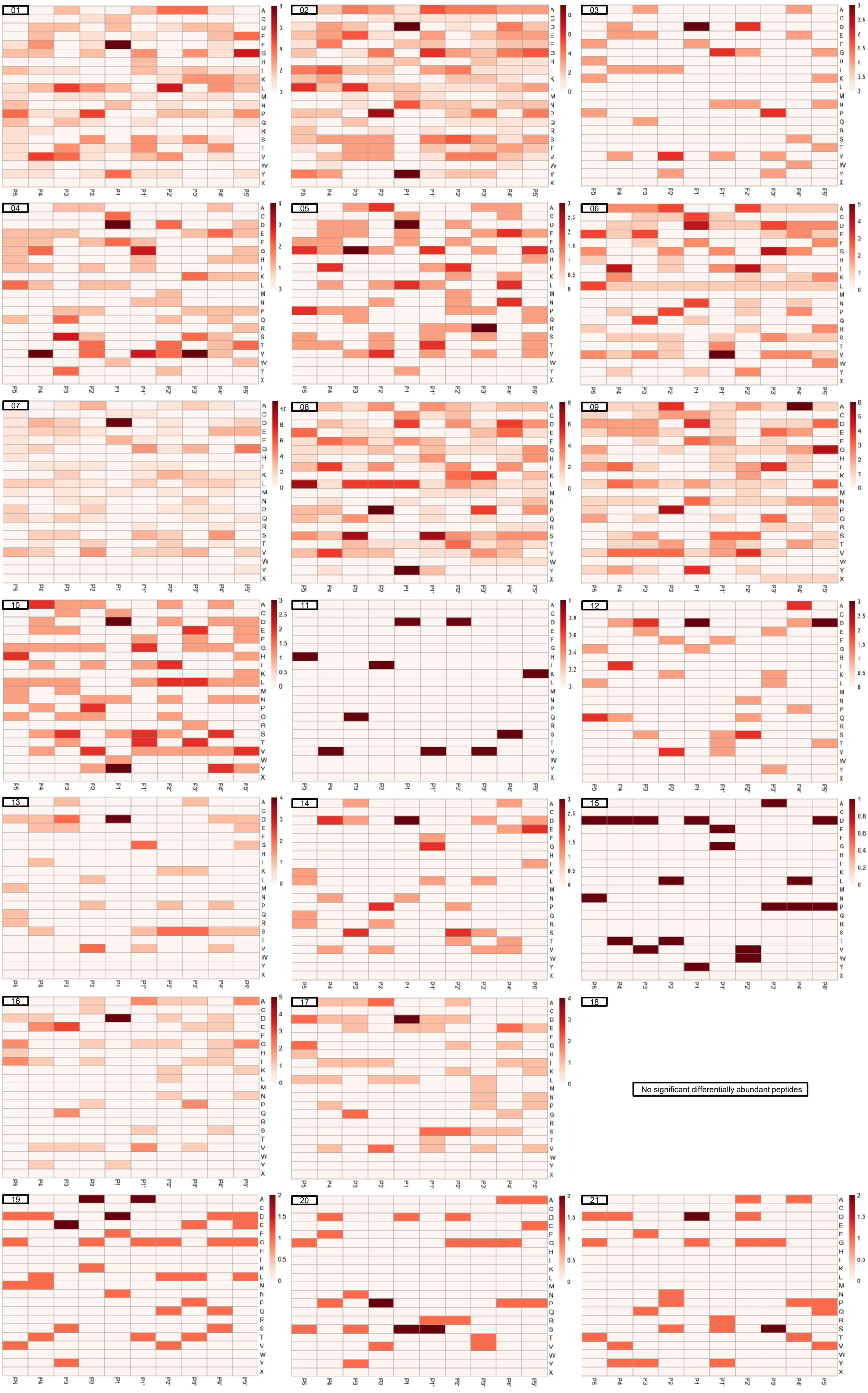

Supplemental figure 5: Heatmap depiction of differentially upregulated cleavage patterns per fraction in nigericin treatment compared to LPS alone utilizing adjusted p-value cutoff of 0.05.
Positional occurrence of amino acids enriched in nigericin compared to LPS treatment alone, calculated by LIMMA differential abundance analysis utilizing adjusted p-value based on semi-specific peptides. Positions displayed as N-terminal (P1-P5) and C-terminal (P1'-P5') of cleavage site between P1 and P1'. Amino acids are stated as one-letter code and fraction number is displayed in top-left box of each heatmap.

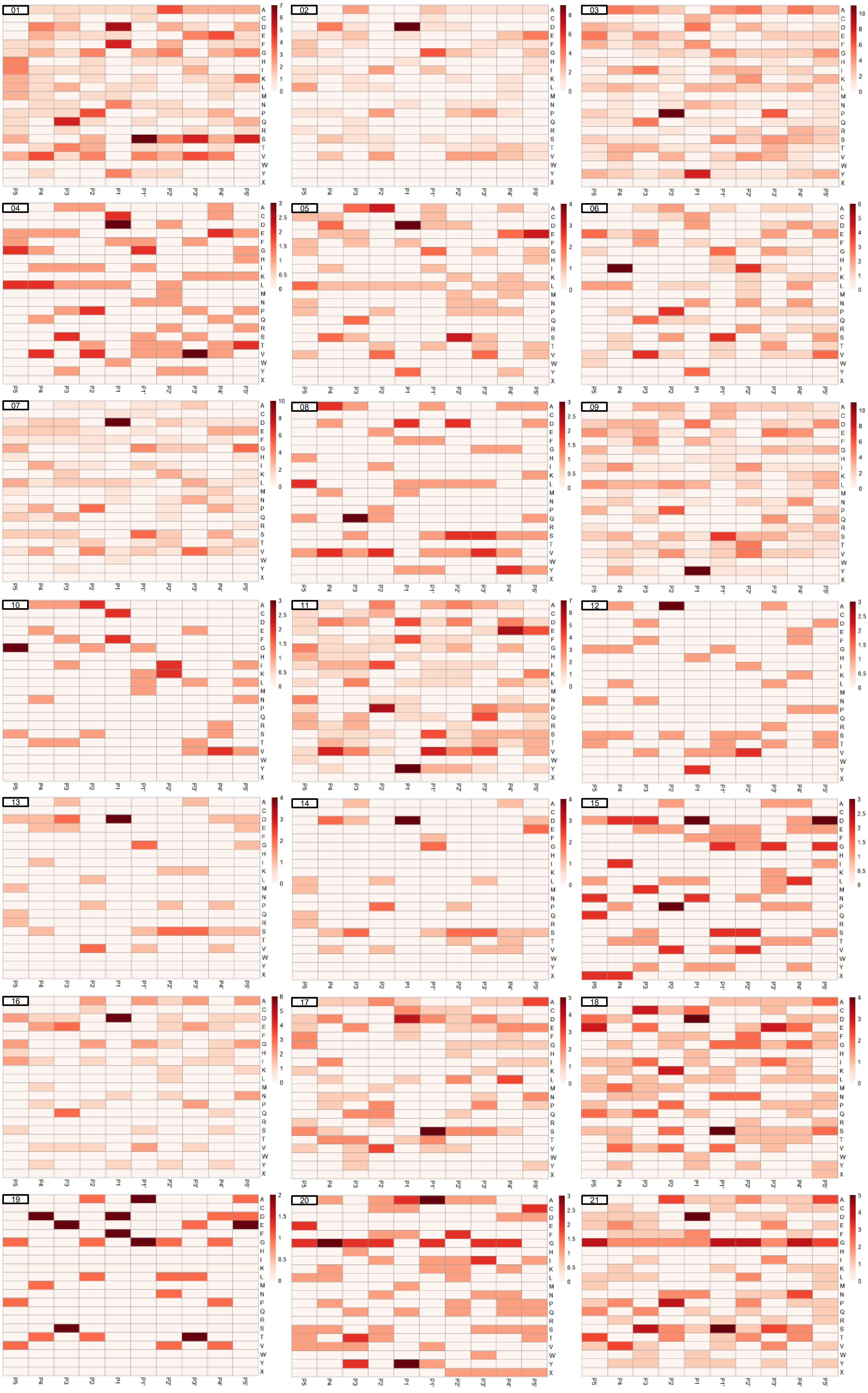

Supplemental figure 6: Heatmap depiction of differentially upregulated cleavage patterns per fraction in VbP treatment compared to LPS alone utilizing adjusted p-value cutoff of 0.05.
Positional occurrence of amino acids enriched in VbP compared to LPS treatment alone, calculated by LIMMA differential abundance analysis utilizing adjusted p-value based on semi-specific peptides. Positions displayed as N-terminal (P1-P5) and C-terminal (P1'-P5') of cleavage site between P1 and P1'. Amino acids are stated as one-letter code and fraction number is displayed in top-left box of each heatmap.

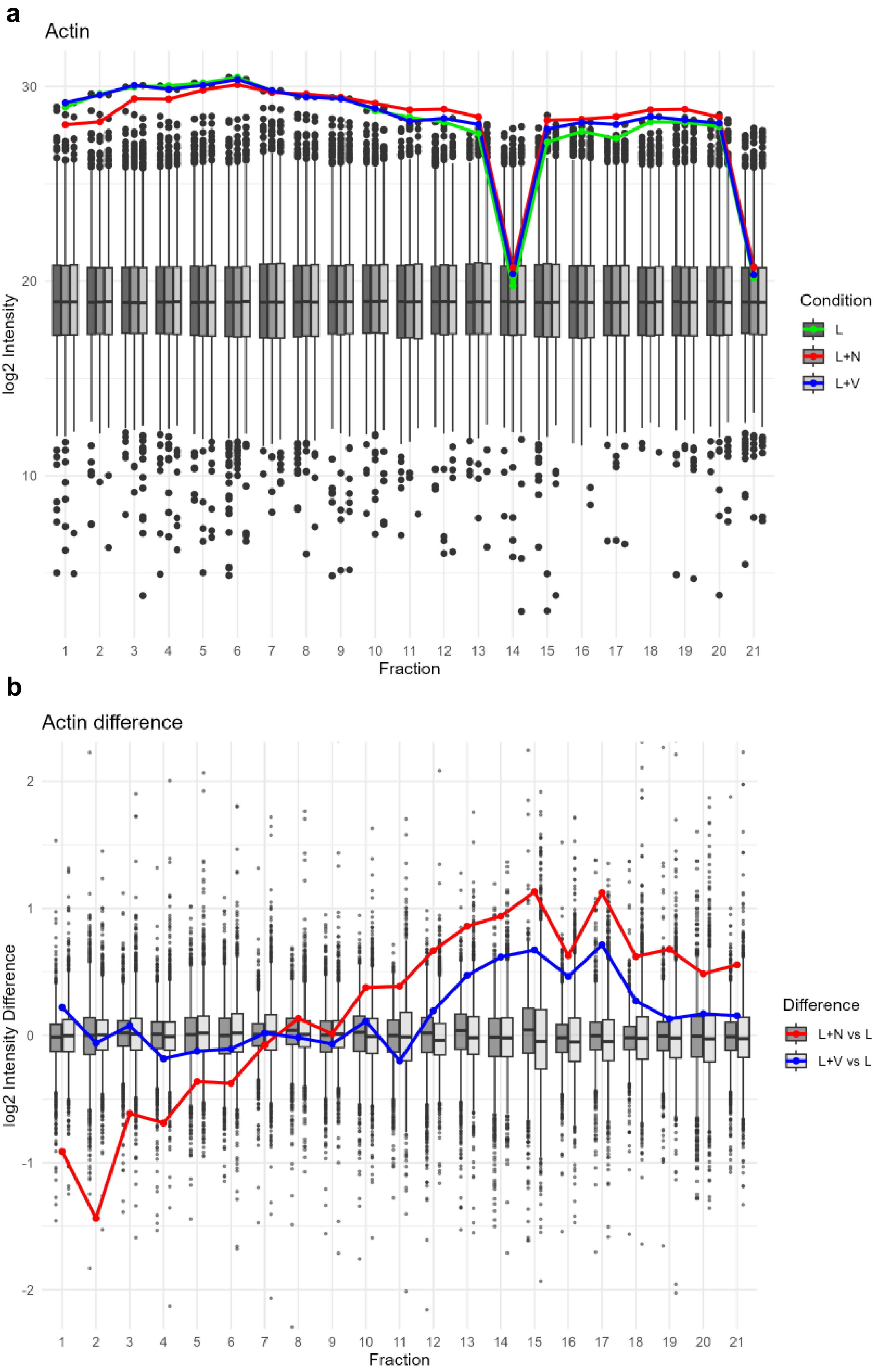

Supplemental figure 7: Actin log2 intensity across SEC fractions.
(**a**) Log2 intensity of actin depicted as line graph for the conditions LPS (L, green), nigericin (L+N, red) and VbP (L+V, blue) in front of boxplot background illustrating the range of abundances for all detected proteins for the conditions LPS (L, dark grey), nigericin (L+N, grey) and VbP (L+V, light grey). (**b** Log2 intensity differences of all detected proteins were calculated by subtracting the log2 intensity values of the LPS condition from those of the nigericin and VbP conditions, per protein and per fraction, respectively. Log2 intensity difference of actin depicted as a line graph for the calculation of nigericin - LPS (L+N vs L, red) and VbP - LPS (L+V vs L, blue) in front of the box plot depiction of the difference range all detected proteins.

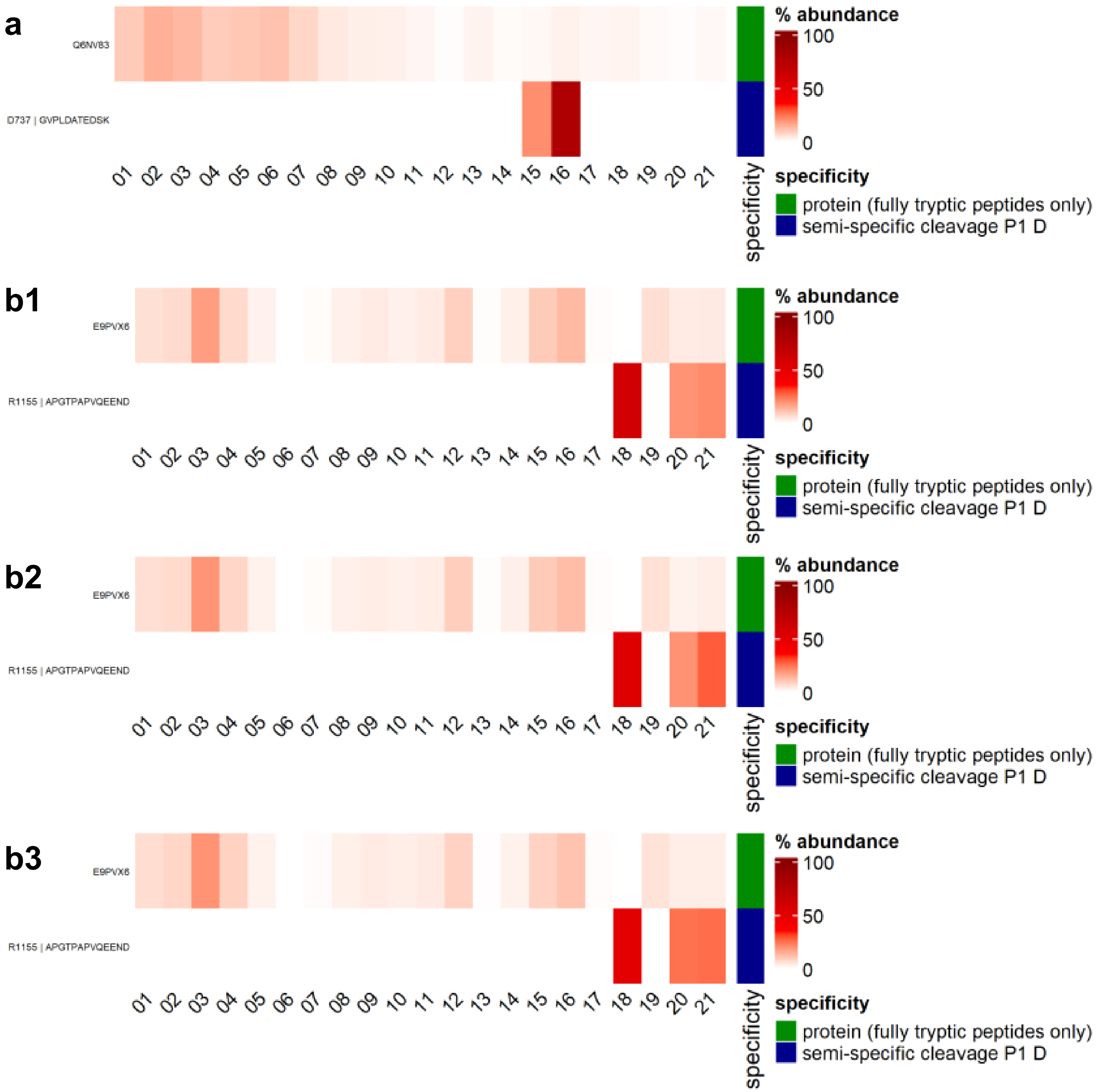

Supplemental figure 8: Exemplary protein and aspartate-cleaved peptide percentage distribution across SEC fractions. (**a**) Heatmap depiction of percentage distribution U2 snRNP-associated SURP motif-containing protein (Q6NV83) and semi-specific peptides, exemplary for nigericin condition. (**b1-b3**) Heatmap depiction of percentage distribution of proliferation marker protein Ki-67 (P17427) and semi-specific peptide in LPS alone condition (**b1**), nigericin condition (**b2**) and VbP condition (**b3**). (**c)** Raw intensity values were summed per row and percentage contribution per fraction is illustrated by red color gradient. Semi-specific peptides with aspartate (D) in P1 are marked as blue and protein, which only consist out of fully tryptic peptides in green.

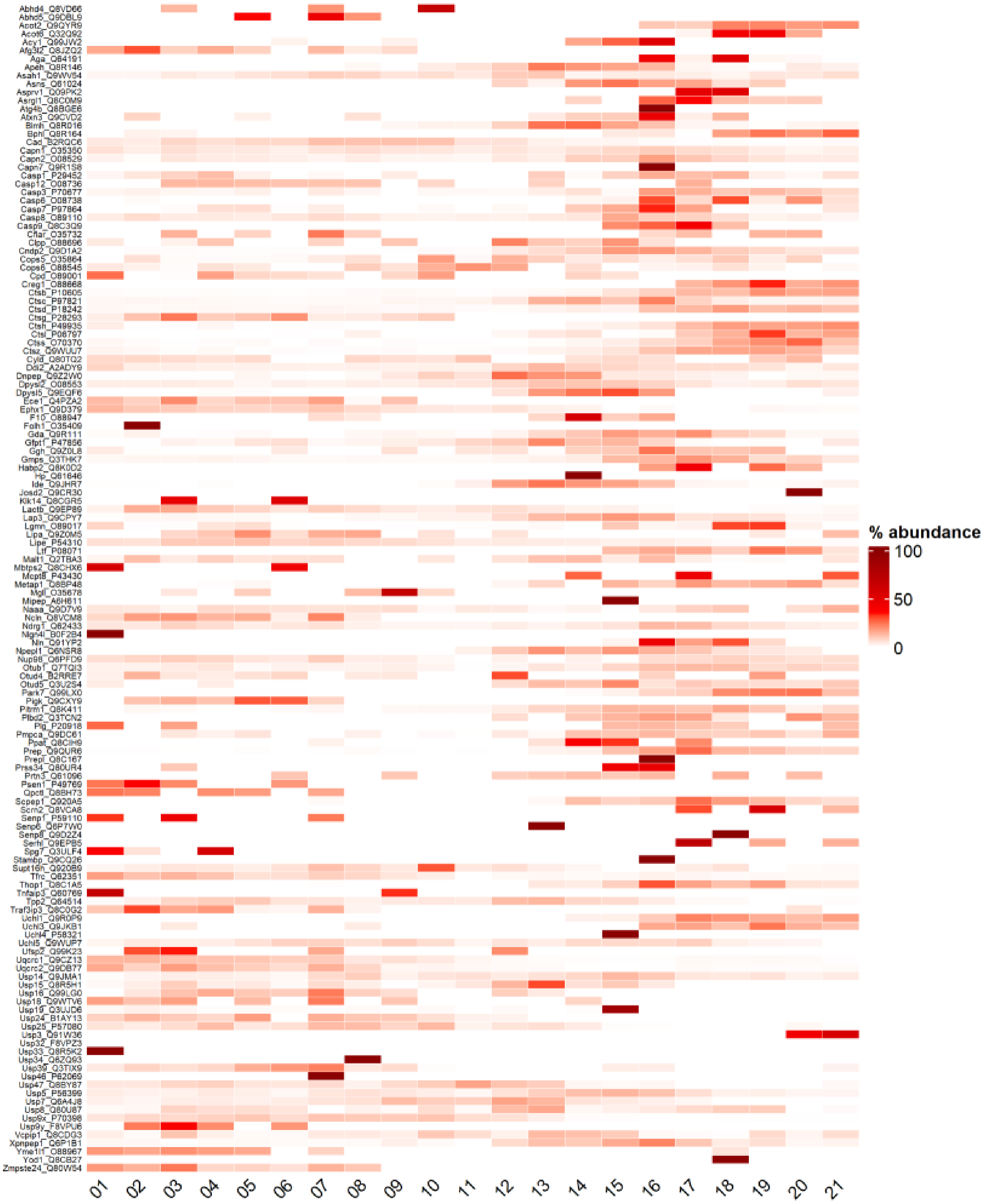

Supplemental figure 9: Percentage distribution of all detected proteases across SEC fractions excluding proteasome, ADAMs, MMPs and DPPs.

Heatmap depiction of percentage distribution of detected proteases. Raw intensity values were summed per row and percentage contribution per fraction is illustrated by red color gradient from 0-100%. Rows named using the combination of to gene name and Uniprot ID of respective protease, fraction number depicted as y-axis.

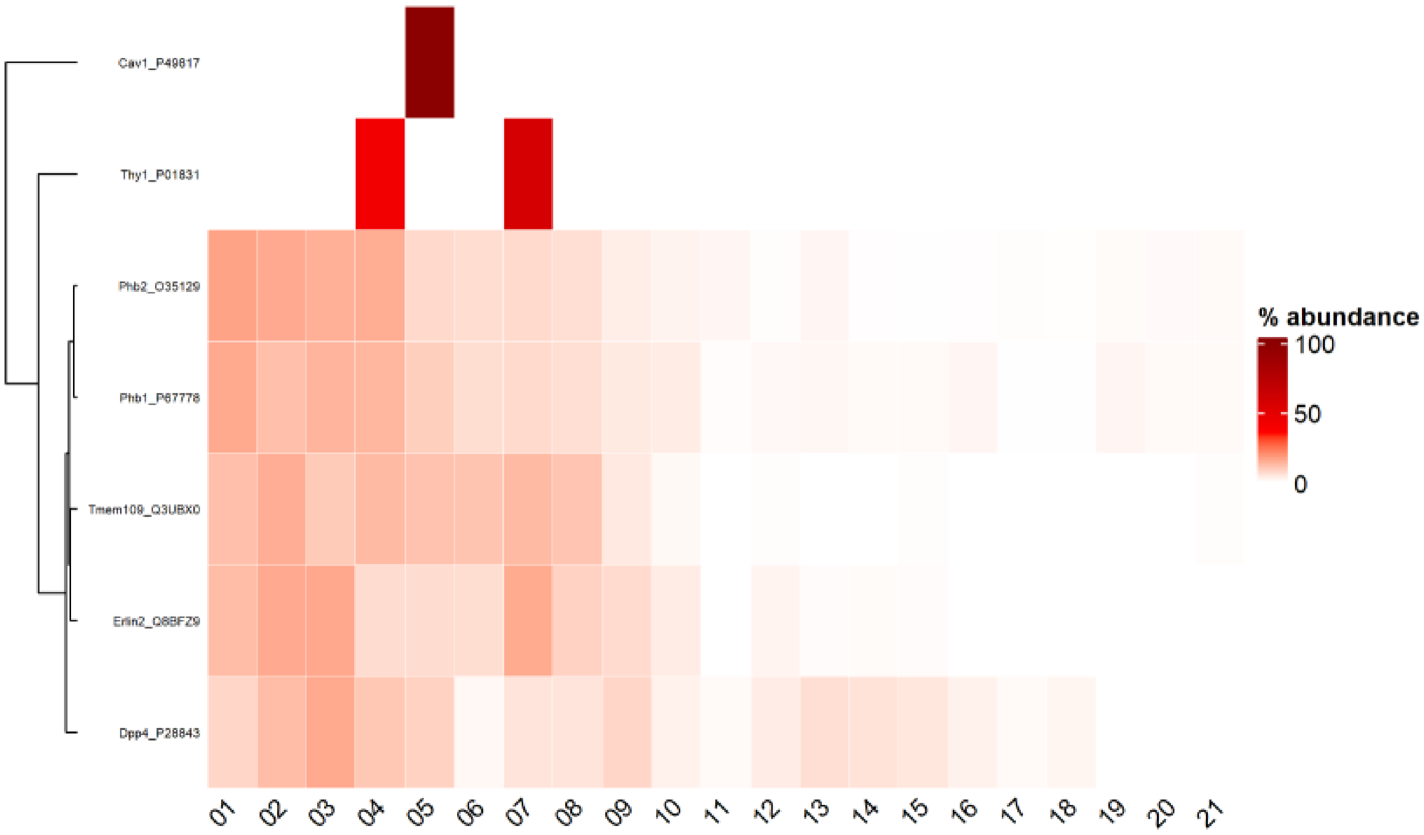

Supplemental figure 10: Percentage distribution of potential lipid raft components across SEC fractions
Heatmap depiction of percentage distribution of caveolin-1 (Cav1), thy-1 membrane glycoprotein (Thy-1), prohibitin-2 (Phb2), prohibitin (Phb1), transmembrane protein 109 (Tmem109), erlin-2 and DPP-4. Raw intensity values were summed per row and percentage contribution per fraction is illustrated by red color gradient from 0-100%. Rows named using the combination of to gene name and Uniprot ID of respective protein, fraction number depicted as y-axis.

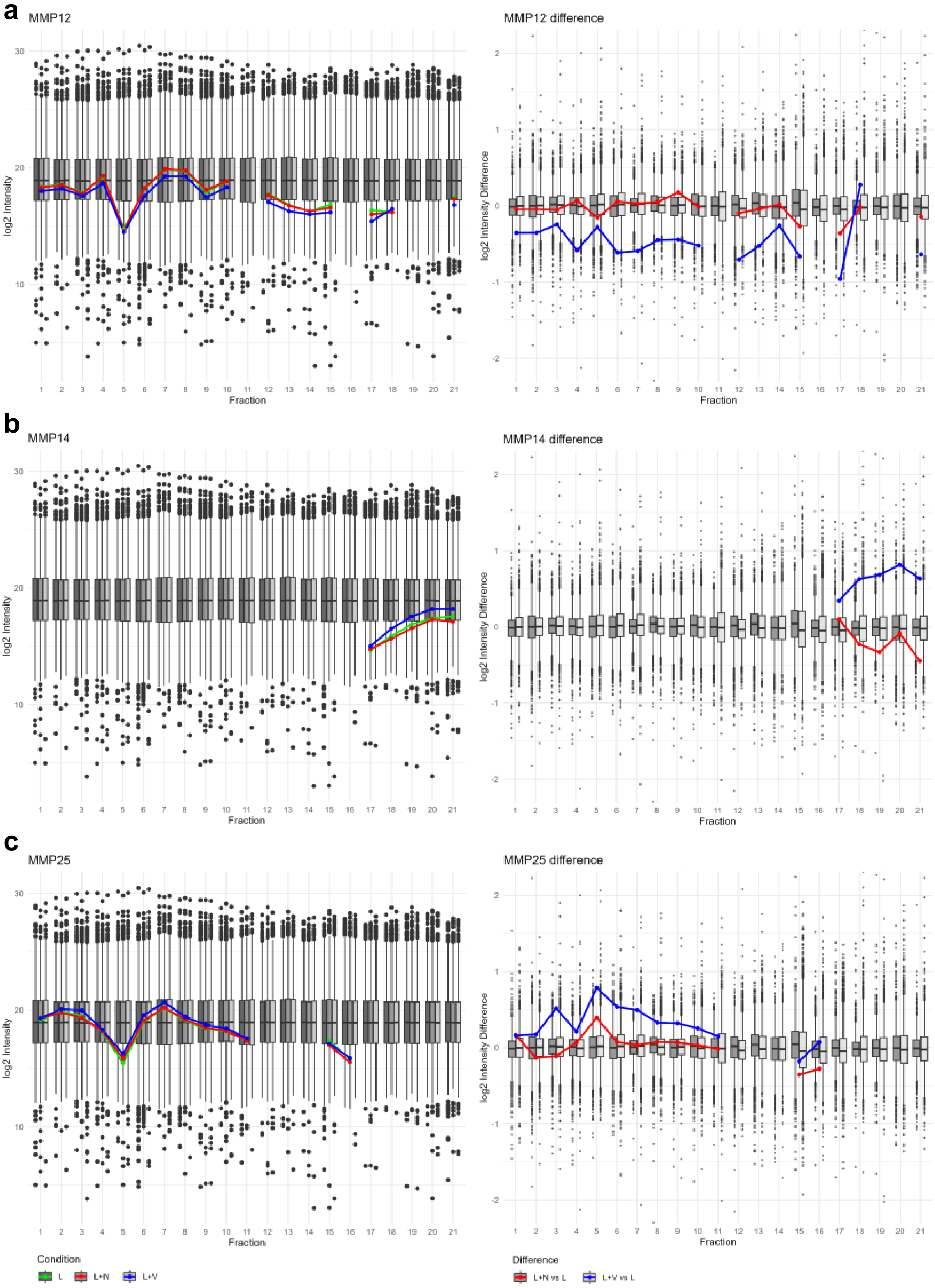

Supplemental figure 11: Differential abundance of MMPs according to log2 intensity distribution and differences across SEC fractions.

(**left**) Log2 intensity distribution of Mmp12 (**a**), Mmp14 (**b**) and Mmp25 (**c**) depicted as line graph for the conditions LPS (L, green), nigericin (N, red) and VbP (V, blue) in front of boxplot background illustrating the range of abundances for all detected proteins for the conditions LPS (L, dark grey), nigericin (N, grey) and VbP (V, light grey). (**right**) Log2 intensity differences of all detected proteins were calculated by subtracting the log2 intensity values of the LPS condition from those of the nigericin and VbP conditions, per protein and per fraction, respectively. Log2 intensity difference of Mmp12 (**a**), Mmp14 (**b**) and Mmp25 (**c**) depicted as a line graph for the calculation of nigericin - LPS (N vs L, red) and VbP - LPS (V vs L, blue) in front of the box plot depiction of the difference range all detected proteins.
